# The effectiveness of GAI-assisted teaching methods in medical education: a systematic review and meta-analysis

**DOI:** 10.3389/fpubh.2026.1813108

**Published:** 2026-04-22

**Authors:** Xingming Ma, Xianting Liu, Haojie Sun

**Affiliations:** 1 School of Social Work and Health Management, Xihua University, Chengdu, China; 2 School of Food and Biological Engineering, Xihua University, Chengdu, China

**Keywords:** artificial intelligence, effectiveness, health education, medical curriculum, medical education, medical students, meta-analysis

## Abstract

**Objective:**

The study evaluated the effectiveness of generative artificial intelligence (GAI)-assisted teaching methods on the medical educational outcomes.

**Methods:**

Following the PRISMA guidelines, a systematic search was conducted for literature on AI-assisted educational interventions in medical education (e.g. clinical, nursing and dentistry medicine). PROSPERO registration number was CRD420251173150. Meta-analyses of the outcomes were performed using the Review Manager 5.4. Heterogeneity was evaluated using the *I*^2^ statistic and Cochran's *Q* test. A forest plot, Egger's test and the trim-and-fill method were used to evaluate publication bias and robustness.

**Results:**

A total of 5,764 publications was initially retrieved, of which 78 studies involving 3,635 medical students in the GAI-assisted teaching group and 3,931 medical students in the control group were included. The pooled results revealed that GAI-assisted teaching significantly improved academic performance in terms of both knowledge (SMD = 0.95, 95% CI: 0.72–1.18, *p* < 0.05) and practical (SMD = 1.48, 95% CI: 1.20–1.77, *p* < 0.05) scores, compared to the control group. Additional benefits included improved student satisfaction (SMD = 1.52, 95% CI: 1.01–2.02, *p* < 0.05), self-efficacy in learning (SMD = 0.75, 95% CI: 0.17–1.32, *p* < 0.05), learning initiative (SMD = 1.20, 95% CI: 0.10–2.30, *p* < 0.05), self-directed learning ability (SMD = 1.25, 95% CI: 0.81–1.69, *p* < 0.05), clinical thinking ability (SMD = 1.18, 95% CI: 0.86–1.50, *p* < 0.05) and analytical and problem-solving skills (SMD = 1.53, 95% CI: 0.77–2.29, *p* < 0.05).

**Conclusions:**

The results showed that the GAI-assisted teaching could improve efficiently various aspects of education outcomes for medical students, including academic performance, self-efficacy and initiative in learning, and skills development. In future, policymakers should consider integrating artificial intelligence into teacher training and medical curriculum design to improve learning outcomes.

## 1 Introduction

Medical education involves advanced academic programs in clinical, nursing and other-related health sciences, providing students with the opportunity to develop their clinical skills and establish their professional identity (1). Over the years, the curriculum for medical majors, including those studying clinical medicine, dentistry and nursing, has been reformed to accommodate a variety of evolving disciplines and an exploding scientific knowledge of the medical sciences to prepare as a competent clinical physician and nurse of the 21st century (2). Traditional medical pedagogical strategies have relied on lecture, simulation-based instruction supplemented with multimedia resources and clinical demonstrations. These teaching approaches limit meaningful interaction, fail to equip students adequately with essential clinical competencies, and often reduce learners to passive recipients of medical knowledge, which ultimately reduce their engagement and skill development throughout the learning process (3). In light of the limitations of this traditional instructional approach, there is a need to develop transformative strategies in medical and health education.

Artificial intelligence (AI) is a broad discipline that can be defined as “a system that can analyze external information, learn from it, and apply this knowledge to achieve a specific aim in a flexible, adaptive manner” (1). The 21st century has witnessed rapid advances in artificial intelligence, which has significantly impacted many industries such as the economy, entertainment, manufacturing, healthcare, medicine, and education (1). The exponential advancement of AI is a key driver of transformation in healthcare and medicine, which is rapidly empowering healthcare services and medical education, from clinical decision support to patient education (4). A 48-country cross-sectional survey has captured widespread enthusiasm among medical students for AI-driven healthcare tools, with strong interest for more AI-focused learning opportunities (5).

Generative artificial intelligence (GAI) is a subset of AI technology that uses large language models to create diverse content through iterative learning from extensive datasets, which is a significant innovation with applications in many fields (6). GAI uses algorithms to analyze existing data, such as images and text, and then generate new content (1). GAI systems such as ChatGPT, DeepSeek, and Google Gemini have demonstrated remarkable capabilities in the medical sciences, including a broader understanding, higher-order thinking, and problem-solving skills (7). These GAI systems have achieved performance levels comparable to those of senior medical students in various licensing examinations (7–9). In the field of medical education, AI-driven tools offer remarkable potential to boost learning efficacy, from producing novel instructional content and designing immersive simulation modules, to building digital patient cases and delivering real-time, individualized feedback and progress assessments (10).

Artificial intelligence can recognize individual differences in students' levels of knowledge and thus generate a tailored, precise education (1). The integration of GAI in medical education is transforming traditional teaching methods, with significant potential to enhance learning outcomes, accelerate skill acquisition, and improve student engagement. The studies have shown that GAI-assisted platforms can improve exam scores by up to 25% and reduce skill acquisition time by 40% (11). A meta-synthesis on the potential applications of ChatGPT suggested that GAI could be used to improve educational efficiency, assist with self-development, enhance communication skills, create and expand the curriculum, and improve the learning environment (12). Seven studies exploring the use of ChatGPT in stomatology education showed promising effectiveness in enhancing knowledge acquisition and diagnostic skills, suggesting GAI may serve as a viable support tool in teaching settings (13). A subsequent study showed that educational interventions using AI-assisted teaching methods could improve medical students' clinical practice skills (14). Nevertheless, certain studies demonstrated that GAI-assisted teaching methods were not effective in enhancing academic performance. A comparative effectiveness evaluation involving 11 eligible studies revealed no significant difference in knowledge acquisition scores between the groups of using GAI-based and traditional teaching approaches (15).

Accordingly, the empirical evidence supporting the effectiveness of GAI-based teaching methodologies in medical education remains inconclusive. This meta-analysis aimed to systematically evaluate the effectiveness of GAI-assisted teaching approaches in the medical education disciplines of clinical medicine, dentistry and nursing by systematically searching English databases and Chinese databases for literature on AI-assisted educational interventions in medical education over the past 20 years.

## 2 Methods

### 2.1 Study design

This meta-analysis and systematic review were performed in strict accordance with the preferred reporting items for systematic reviews and meta-analysis (PRISMA) in Appendix 1 (16). Ethical approval and patient consent were not required as all analyses were based on previously published studies. This study was registered on PROSPERO (CRD420251173150) and the full protocol can be accessed on PROSPERO's website.

### 2.2 Search strategy

A comprehensive literature search was performed by two researchers across multiple electronic databases, including the English databases of PubMed, Web of Science, ScienceDirect, Embase, and the Cochrane Library, as well as the Chinese electronic databases of CNKI, Wanfang, CQVIP, and CBM. The search period spanned from 1 January 2005 to 14 November 2025. The specific set of MeSH terms or title or keywords or abstract was used to search the English databases, and the topic or title or keywords or abstract was used to search the Chinese databases. The detailed search strategy is provided in Appendix 2.

### 2.3 Study selection criteria

The PICOS (Population, Intervention, Comparison, Outcome and Study design) framework was used to determine the inclusion criteria for the studies. The following criteria were applied: (a) The studies were published in peer-reviewed English and Chinese journals. (b) The participants were undergraduate medical students, including those students in clinical medicine, nursing and dentistry. (c) The experimental group received GAI-assisted teaching methods. (d) The control group received traditional or other standard teaching approaches without GAI assistance. (e) The curricula covered medical and/or biomedical disciplines. (f) The outcomes presented as data included primary outcomes such as final knowledge exam scores or/and practical assessment scores. (g) The studies were two-group controlled (non-randomized and/or randomized). (h) All the above studies were conducted between 1 January 2002 and 14 November 2025.

### 2.4 Study exclusion criteria

Studies were excluded based on one of the following criteria: (a) not relevant to the topic of this meta-analysis (i.e., not employing GAI-assisted teaching methods); (b) retracted articles, reviews, editorials, conference abstracts, case reports, or book chapters; (c) duplicate publications or studies with overlapping participant data; (d) insufficient data for outcome calculation or lack of quantitative outcomes related to effectiveness; or (e) publications in languages other than English or Chinese.

### 2.5 Data extraction

Two investigators independently extracted the data using an electronic form (Microsoft Excel), which included study characteristics (authors, publication date, and country), participant details (sample size, grade, course and major), GAI-assisted teaching intervention (tools related to GAI and learning duration), and outcomes. The primary outcomes were knowledge scores (KS) or practical scores (PS) after exams. The teaching satisfaction (TS) and skill assessment scores or frequency such as learning self-efficacy (LSE), learning initiative (LI), Self-directed learning ability (SLA), clinical thinking ability (CTA), analytical and problem-solving skills (APS), or critical thinking skills (CTS) of students were considered as the secondary outcomes.

### 2.6 Quality assessment

The Cochrane risk of bias 2 (RoB2 v9) tool was used to assess the quality of the randomized controlled trials. The evaluation criteria included the following five domains and an assessment of overall bias: randomization process, deviation from intended interventions, missing outcome data, measurement of the outcome, and selection of the reported result. For non-randomized studies, the MINORS scale was used. The evaluation criteria included the following 12 domains and an assessment of overall bias: research objectives, study population, data collection, appropriate endpoints, unbiased assessment, matching follow-up period, loss to follow-up of < 5%, sample size calculation, adequate control group, contemporary groups, baseline characteristics, statistical analysis. Any disagreements were resolved by the corresponding author.

### 2.7 Publication bias assessment and sensitivity analysis

A forest plot was visually inspected to assess potential publication bias, an Egger's test and Duval and Tweedie's Trim-and-fill method was performed to statistically evaluate asymmetry and robustness of the research results. Sensitivity analysis was conducted using the one-study-removed method to evaluate the impact of each trial on the overall effect size.

### 2.8 Data synthesis

Data synthesis was performed in Microsoft Excel, and pooled effect sizes were estimated for all outcomes. Continuous variables were summarized as standardized mean differences (SMD) and 95% confidence intervals (CI), while dichotomous variables were summarized as odds ratios (OR) and 95% CIs. Heterogeneity was measured by *I*^2^, with values of *I*^2^ = 30%−75% or *I*^2^ > 75% representing moderate and high heterogeneity, respectively (17). The meta-analysis used a random-effects model for the pooled effect sizes in cases of moderate or high heterogeneity, and with statistical significance defined as *p* < 0.05. The Cochrane's Review Manager (RevMan) 5.4 was used for statistical analysis. The Gpower 3.1 was used to conduct a power analysis for meta-analysis (18).

## 3 Results

### 3.1 Database searching and selection

**Figure 1:**
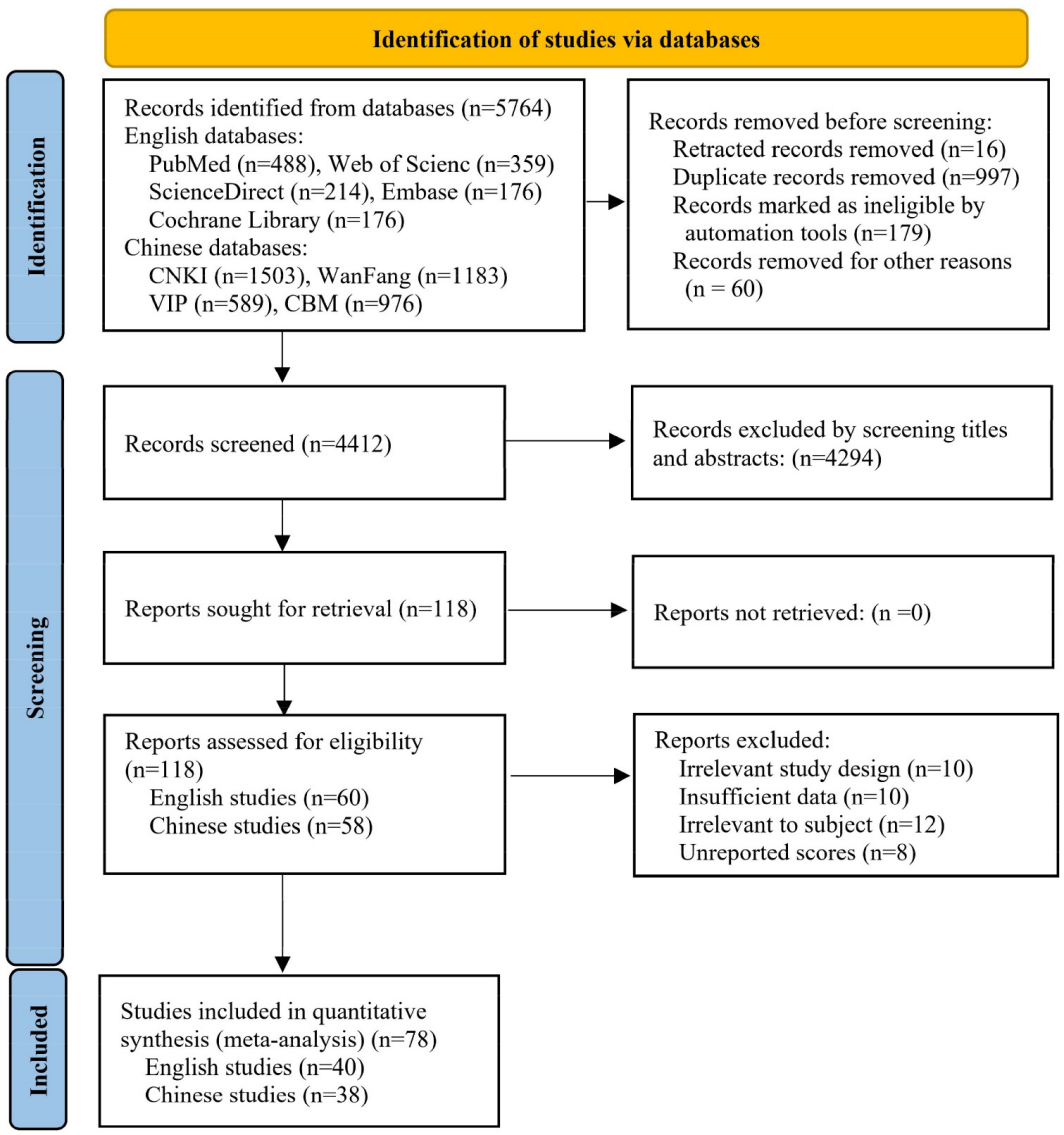
The PRISMA selection flowchart for the included studies.

Figure 1 showed the PRISMA selection flowchart. This search yielded a total of 5,764 studies. After removing duplicates, retracted studies and ineligible studies, 4,412 titles and abstracts were screened. According to the titles and abstracts, 4,294 studies were excluded as they were irrelevant to the subject or lacked quantitative measurement of the scores. Following a detailed examination of the full texts, a further 40 studies were excluded. Ultimately, this systematic review included a total of 78 eligible trials.

### 3.2 Study and participant characteristics

The meta-analysis included 61 RCTs and 17 observational non-randomized controlled trial with a total of 7,655 students, comprising 3,931 participants in the control groups and 3,635 participants in the GAI- assisted intervention groups (19–96). The study population primarily consisted of medical students of undergraduate including clinical medical students (52 studies, 66.7%), nursing students (17 studies, 21.8%), and dental students (7 studies, 9.0%), with two studies of occupational therapy students. All included studies were published between 2016 and 2025, with a predominant representation from mainland China institutions (51 studies, 65.4%). Out of these 78 studies, only 40 was published in English, and 38 were published in Chinese. The ChatGPT (20 studies, 25.6%) and the self-developed AI learning platform (22 studies, 28.2%) was the primary GAI tool utilized for learning, and there were also 14 studies that did not report the names of the AI tools used. 52 studies (66.7%) implemented interventions exceeding 1 weeks. 17 studies (21.8%) implemented interventions only single-session intervention, and 9 studies (11.5%) were not to report the time of implemented interventions. Detailed baseline characteristics of all included studies are presented in Table 1.

**Table 1:** The baseline characteristics of the included studied.

Author/ Reference	Design	Region	Course	Population	Comparison Exp. vs. Con.	Learning duration	Number Exp. vs. Con.	Quality	Outcome
Kalam et al. (19)	RCT	USA	Basic medical sciences	Medical students	Learning with ChatGPT-4.0 vs. Conventional Institutional resources with lecture materials, e-textbooks	One lesson	10 vs. 11	Low risk	KS, TS
Aneesh et al. (20)	Non-RCT	India	Physiology	Medical students	Learning with Perplexity AI vs. Conventional learning with textbooks	6 h	103 vs. 103	High quality	KS, TS
Chen (21)	RCT	China	Systemic anatomy	Medical students	AI-PLP based on Coze platform vs. Traditional lecture-based teaching	12 weeks	20 vs. 20	Low risk	KS, TS
Zeng et al. (22)	RCT	China	Ophthalmology	Medical students	AI-assisted learning with ChatGPT vs. Conventional materials and internet	One lesson	72 vs. 70	Low risk	KS
Huang et al. (23)	RCT	China	Dental clinical operation	Dental students	Learning with ChatGPT-3.5 vs. Conventional teaching with video	One week	94 vs. 93	Some concerns	KS, PS
Ergezen Sahin et al. (24)	RCT	Turkey	Chronic low back pain rehabilitation	Physical therapy student	AI-assisted PBL with ChatGPT-4.0 vs. Traditional instructor-led PBL	2 weeks	19 vs. 16	Some concerns	KS, PS, LSE, LI
Li et al. (25)	RCT	China	Medical biochemistry	Medical students	Kimi Chat 2.0-assisted CBL learning vs. Traditional case-based learning methods	One lesson	39 vs. 40	Some concerns	KS
Han et al. (26)	RCT	Korea	Mechanical ventilation nursing	Nursing students	Chatbot (LandBot.io) educational and video lectures vs. Traditional Video lectures program and video lectures	One lesson	31 vs. 29	Low risk	KS, PS, TS, LSE, CTA
Molu (27)	RCT	Turkey	Neonatal resuscitation	Nursing students	AI-based learning using ChatGPT vs. Traditional instruction with PPT	4 weeks	35 vs. 35	Low risk	KS
Kejingyun and Mingjun (28)	RCT	China	Nursing education	Nursing students	LLM-assisted problem-based learning vs. Traditional PBL	8 weeks	50 vs. 50	Low risk	KS, CTA, CTS
Wu et al. (29)	RCT	China	Hepatobiliary surgery	Medical students	ChatGPT-based blended teaching vs. Traditional teaching methods	One semester	31 vs. 30	Low risk	KS, PS, TS, LSE
Mayor-Silva et al. (30)	RCT	Spain	Occupational risk prevention law	Nursing students	Learning with ChatGPT-3.5 vs. Traditional teaching methods	One lesson	69 vs. 68	Some concerns	KS, APS
Tseng et al. (31)	Non-RCT	Taiwan	Writing case reports and seminars	Nursing students	Writing with ChatGPT and Copilot-assisted learning vs. Traditional writing instruction learning	18 weeks	102 vs. 101	Medium quality	KS
Höhne et al. (32)	RCT	Germany	Lung ultrasound and focused assessment with sonography for trauma (FAST)	Medical students	AI-assisted blended e-learning using the ScanLab app and AI feedback vs. Traditional instructor-led, hands-on ultrasound workshop	4 h	25 vs. 25	Some concerns	KS, PS
Coelho et al. (33)	RCT	Brazil	Pulpal and periapical diagnosis in endodontics	Dental students	Chatbot delivered via telegram vs. Expository interactive lecture by endodontist	One lesson	11 vs. 11	Some concerns	KS
Du et al. (34)	RCT	China	Early pediatric orthodontics	Dental students	AI-assisted (DeepSeek-R1) flipped classroom vs. Traditional lecture-based teaching	NR	33 vs. 33	Some concerns	KS, PS, SLA
Qin et al. (35)	RCT	China	Oral mucosal diseases	Dental students	AI-based (ChatGLM platform) personalized teaching strategy vs. Traditional lecture-based teaching	One semester	30 vs. 30	Some concerns	KS, PS, LI
Zhu et al. (36)	RCT	China	Pediatric clinical Practice	Medical students	AI-assisted (kimi) PBL and CBL teaching vs. Traditional PBL and CBL teaching	One semester	33 vs. 35	Some concerns	KS, PS, TS
Fu et al. (37)	RCT	China	Diagnostics	Medical students	Learning with Huazhi-Yihui AI-assisted diagnostic teaching platform vs. Traditional case-based teaching	One semester	30 vs. 30	Low risk	KS, PS, TS, SLA, CTA, CTS
Fan et al. (38)	Non-RCT	China	Pathogenic biology and immunology	Medical students	Blended teaching based on AI knowledge graph vs. Blended teaching based only on the online resources	One semester	57 vs. 57	Medium quality	KS
Liu et al. (39)	RCT	China	Hand and foot surgery	Medical students	AI-assisted teaching vs. Conventional teaching (teacher-dominated lectures, student observation followed by hands-on practice, on-site teacher guidance).	NR	30 vs. 30	Low risk	KS, PS, TS, APS
Zhu et al. (40)	RCT	China	Pathology	Medical students	AI-assisted (ChatAI and DeepSeek) diagnostic teaching vs. Conventional routine pathology teaching	One semester	59 vs. 61	Some concerns	KS, PS, TS, LI, CTA
Shi et al. (41)	Non-RCT	China	Neurology	Medical students	AI-empowered (DeepSeek, DouyinDoubao, KedaXunfei, and ZhipuQingyan) smart learning vs. Traditional lecture-based teaching	One semester	120 vs. 118	Medium quality	KS, TS
Zheng et al. (42)	RCT	China	Radiologic imaging	Medical students	Learning with AI-assisted diagnosis platform vs. Traditional teaching (observed case and analysis, case discussion, wrote reports)	8 weeks	51 vs. 51	Some concerns	KS, PS, TS, CTA, APS
Yu et al. (43)	RCT	China	Thoracic surgery	Medical students	AI-assisted micro-lecture and flipped classroom vs. Traditional lecture-based teaching	NR	53 vs. 53	Low risk	KS, PS, TS, CTA, CTS
Liu et al. (44)	Non-RCT	China	Pain medicine	Medical students	AI combined with flipped teaching vs. Flipped teaching	One semester	42 vs. 43	Medium quality	KS, TS, SLA
Zhu et al. (45)	RCT	China	Pathophysiology	Nursing students	AI-empowered BOPPPS teaching vs. Traditional lecture-based teaching	One semester	87 vs. 83	Low risk	KS, TS, SLA
Feng et al. (46)	RCT	China	Nephrology	Medical students	AI-assisted (ChatGPT) BOPPPS teaching vs. Traditional lecture-based teaching	One semester	28 vs. 28	Some concerns	KS, TS, CTA
Wang et al. (47)	RCT	China	Orthopedics	Medical students	generative AI-assisted teaching vs. Traditional lecture-based teaching	4 weeks	77 vs. 77	Low risk	KS, PS, SLA
Zhang et al. (48)	RCT	China	Thyroid and breast surgery	Medical students	AI-assisted (DeepSeek R1) personalized teaching vs. Traditional lecture-based teaching	4 weeks	40 vs. 40	Low risk	KS, PS, APS
Liang et al. (49)	RCT	China	Respiratory medicine	Medical students	Learning with intelligent case-based online system vs. Traditional lecture-based teaching	One semester	42 vs. 42	Low risk	KS, SLA, CTA, APS
Liang et al. (50)	RCT	China	Neurosurgery	Medical students	ChatGPT-assisted BOPPPS teaching vs. Traditional teacher-led clerkship	NR	58 vs. 57	Low risk	KS, TS
Cheng et al. (51)	RCT	China	Cardiology	Medical students	ChatGPT-assisted instruction learning vs. Traditional lecture-based teaching	2 weeks	31 vs. 35	Some concerns	KS
Wang et al. (52)	RCT	China	Radiologic nursing	Nursing students	AI-assisted (DeepSeek) SPOC and flipped classroom vs. Traditional lecture-based teaching	2 months	40 vs. 40	Low risk	KS, PS, TS, SLA, LI
Gan et al. (53)	RCT	China	Orthopedics	Medical students	ChatGPT-4.0 assisted learning vs. Traditional Internet search learning	2 weeks	54 vs. 56	Some concerns	KS
Zheng et al. (54)	Non-RCT	China	Cardiovascular diseases	Medical students	AI-empowered scenario-based simulation teaching (ChatGPT-3.5) vs. Traditional teaching with lectures	NR	34 vs. 32	Medium quality	KS, PS, TS, CTA
Wang et al. (55)	non-RCT	China	Medical imaging	Medical students	AISD software based on VDR technique used in practical teaching vs. Traditional teaching with film and PPT software	One semester	41 vs. 43	Medium quality	KS, PS, LSE, SLA
Akutay et al. (56)	RCT	Turkey	Musculoskeletal diseases and nursing care	Nursing	AI-assisted THA case with animated avatar & Mentimeter vs. Instructor-led PPT presentation	One lesson	94 vs. 94	Some concerns	KS, TS
Roganović (57)	Non-RCT	Serbia	Dental pharmacology	Dental students	Learning with ChatGPT-3.5 vs. Learnng with recommended literature	One lesson	13 vs. 47	Medium quality	KS
Bhatia et al. (58)	RCT	India	Anatomical landmarks	Dental students	ChatGPT-assisted learning vs. Traditional lecture and textbook	One lesson	82 vs. 82	Some concerns	KS
Zhao et al. (59)	RCT	China	Surgery	Medical students	AI-assisted (Rain Classroom) blended teaching vs. Traditional lecture-based teaching	4 months	32 vs. 31	Low risk	KS, TS, SLA, CTA
Li et al. (60)	RCT	China	Medical imaging	Medical students	AI-assisted BOPPPS teaching vs. Traditional lecture-based teaching	3 months	30 vs. 30	Low risk	KS, PS
Cui et al. (61)	RCT	China	Medical imaging	Medical students	AI-assisted teaching vs. Traditional lecture-based teaching	2 weeks	40 vs. 40	Some concerns	KS, PS, TS
Ke et al. (62)	Non-RCT	China	Emergency medicine	Traditional Chinese Medicine student	AI-assisted video laryngoscope tracheal intubation simulator training vs. Traditional ordinary video laryngoscope tracheal intubation simulator training	One lesson	42 vs. 38	Medium quality	KS, PS, TS
Feng et al. (63)	RCT	China	Diabetes mellitus	Medical students	Learning with AI electronic simulated patient inquiry software vs. Traditional case-based teaching (bedside inquiry, physical examination, case discussion)	NR	43 vs. 42	Low risk	KS, PS, TS, SLA, CTA, APS
Bai et al. (64)	RCT	China	Parkinson's disease	Medical students	Learning with AI-assisted Parkinson APP vs. Traditional teaching including outpatient consultation, physical examination, history reporting, discussion, and medical record writing.	One month	40 vs. 40	Low risk	KS, PS
Liu et al. (65)	Non-RCT	China	Medical imaging diagnostics	Medical students	Learning with AI-assisted Superstar learning pass system vs. Traditional teaching with Superstar Learning Pass system	NR	46 vs. 45	Medium quality	KS, PS, TS, SLA, LI, CTA, APS
Zhong et al. (66)	RCT	China	Locomotor system diseases	Medical students	learning with AI kinematic system learning software vs. Traditional demonstration teaching	One semester	40 vs. 40	Low risk	KS, PS, TS
Liaw et al. (67)	RCT	Singapore	Sepsis care and interprofessional communication	Nursing students	AI-powered virtual doctor in VR sepsis team training vs. Human-controlled virtual doctor teaching	One lesson	32 vs. 32	Low risk	KS, PS, LSE
Al Kahf et al. (68)	RCT	France	Pulmonology	Medical students	AI-assisted case analysis with DALL-E3 & D-ID animated visuals vs. Traditional teaching with PPT case analysis	6 weeks	104 vs. 255	Some concerns	KS
Zhao et al. (69)	RCT	China	Oncology	Medical students	Watson for oncology platform for case-based learning vs. Traditional case-based learning without Watson for oncology platform	6 weeks	36 vs. 31	Low risk	KS, TS, SLA, LI
Cai et al. (70)	RCT	China	Medical imaging	Medical students	Learning with CBL and AI software (InferViewer) vs. Traditional lecture-based teaching	One lesson	30 vs. 30	Some concerns	KS, PS, TS
Han et al. (71)	Non-RCT	Korea	Electronic fetal monitoring (EFM) nursing	Nursing students	AI chatbot educational program (LandBot.io) vs. Traditional video lecture	2 weeks	30 vs. 31	High quality	KS, TS, LSE, SLA, LI, CTA
Wang et al. (72)	RCT	China	Ophthalmic Nursing	Nursing students	Learning with ophthalmic AI diagnosis and treatment system vs. Traditional lecture-based teaching	One lesson	51 vs. 50	High risk	KS, PS, TS, CTA
Liu et al. (73)	RCT	China	Medical imaging	Medical students	Learning with AI-assisted RIS-PACS system vs. Traditional lecture-based teaching	One year	50 vs. 50	Some concerns	KS, TS
Yang et al. (74)	Non-RCT	China	Echocardiography	Medical students	Learning with AI-assisted echocardiography diagnosis software (Beijing Ande Yizhi) vs. Traditional hands-on teaching	3 weeks	30 vs. 30	Medium quality	KS, PS, TS
Fernández-Alemán et al. (75)	RCT	Spain	Anatomy of the locomotor system	Medical students	Learning with intelligent SIDRA vs. Traditional teaching methodology without i-SIDRA	15 weeks	76 vs. 88	Some concerns	KS
Chang et al. (76)	Non-RCT	Taiwan	Obstetric care	Nursing students	AI-based (ChatGPT, Xmind) GAI-PCC strategy vs. Conventional C-CPTS strategy	5 weeks	33 vs. 33	Medium quality	PS, LSE, CTA, APS
Chun et al. (77)	RCT	Korea	Assistive technology in occupational therapy	Occupational therapy students	AI-powered textbook based on LLama 3.1 vs. Conventional teaching strategy	15 weeks	43 vs. 43	Low risk	PS, TS
Gokkurt Yilmaz et al. (78)	RCT	Turkey	Radiographic diagnosis	Dental students	AI-personalized feedback with ChatGPT-4o vs. Standard correct/incorrect feedback analysis	One month	55 vs. 55	Low risk	PS, TS
Liu et al. (79)	Non-RCT	China	Blood cell morphology	Medical students	AI-powered online learning platform (DeepCyto system) for virtual microscopy vs. Traditional microscope-based teaching	3 h	27 vs. 37	Low risk	PS
Wang et al. (80)	RCT	China	History-taking training	Medical students	learning with AI-generated patients powered by the GPT-4 API vs. Traditional role-playing by instructors	4 weeks	28 vs. 28	Low risk	PS
Dupont et al. (81)	RCT	France	Nephrology-specific OSCE preparation	Medical students	Access to OSCE preparation podcast (NephrOdio) vs. No podcast access (traditional learning methods).	4 weeks	25 vs. 25	Low risk	PS, LSE
Hui et al. (82)	RCT	China	Urology	Medical students	ChatGPT-assisted PBL teaching vs. Traditional PBL teaching	2 weeks	21 vs. 21	Some concerns	PS, CTA, APS
Hassoulas et al. (83)	RCT	UK	Cardiology, neurology, orthopedics, and gastrointestinal medicine within a CBL curriculum	Medical students	Technology-enhanced CBL and GenAI simulated virtual patient platform vs. Conventional CBL teaching	One semester	10 vs. 10	Some concerns	PS
Kestel et al. (84)	RCT	Turkey	History-taking training	Nursing students	AI chatbot-assisted (LINE/web) vs. traditional text reading methods	2 weeks	38 vs. 41	High risk	PS
Uysal Yalçin et al. (85)	RCT	Turkey	Nursing care technologies and use of artificial intelligence in nursing	Nursing students	Individual self-study using ChatGPT teaching vs. Traditional face-to-face lecture.	3 weeks	23 vs. 25	Low risk	PS
Liaw et al. (86)	Non-RCT	Singapore	Clinical deterioration training	Nursing students	AI-enabled (ChatGPT) VR simulation teaching vs. Conventional in-person simulation teaching	3 h	60 vs. 87	Medium quality	PS
Feng et al. (87)	RCT	China	Basic clinical skills	Medical students	Training and assessment via an AI-assisted clinical-skills evaluation system vs. Conventional method for training and assessment	12 weeks	120 vs. 120	High risk	PS, TS, CTA, APS
Gao et al. (88)	RCT	China	Anesthesiology	Medical students	Teaching mode with integrating virtual reality and AI-assisted vs. Traditional lecture-based teaching	NR	30 vs. 30	Some concerns	PS
Yamamoto et al. (89)	Non-RCT	Japan	Medical interview	Medical students	AI-simulated patient interactions teaching with GPT-4 Turbo chatbot vs. Traditional simulation practice	One month	35 vs. 110	Medium quality	PS
Yilmaz et al. (90)	RCT	Canada	Neurosurgical tumor	Medical students	Visuospatial feedback with 3D spatial models vs. Practice alone with no tailored performance feedback	One lesson	31 vs. 30	Low risk	PS
Simsek-Cetinkaya et al. (91)	RCT	Turkey	Breast examination training	Nursing students	AI-assisted Interactive Screen-Based Simulation learning vs. Standard patient simulation learning	One lesson	52 vs. 51	Some concerns	PS, TS
Kang et al. (92)	RCT	China	Coronary artery disease	Medical students	Learning with AI-assisted diagnosis system vs. Traditional PPT-based teaching	One lesson	60 vs. 60	Low risk	PS, TS
Xu et al. (93)	RCT	China	Medical imaging	Medical students	Learning with AI-assisted PACS teaching software vs. Traditional lecture-based teaching	NR	30 vs. 30	Low risk	PS
Yan et al. (94)	RCT	China	Chest CT imaging diagnostics	Medical students	Learning with CT image-assisted detection software and AI-annotated results vs. Traditional lecture-based teaching	2 weeks	40 vs. 40	Low risk	PS, SLA, LI, APS, CTS
Gao et al. (95)	RCT	China	Bone cell morphology	Medical students	Learning with AI-based Marrow Cell Morphology Picture Storage and Transfer System vs. Traditional teaching with multimedia and microscope image interpretation	One lesson	55 vs. 55	Some concerns	PS, TS, CTA
Miao et al. (96)	Non-RCT	China	Emergency nursing	Nursing students	Learning with intelligent medical comprehensive simulation system vs. Traditional skill training	One semester	96 vs. 98	Medium quality	PS, TS, LI, APS

Non-RCT, non-randomized controlled trial; RCT, randomized controlled trial; KS, knowledge scores; PS, practical scores; TS, teaching satisfaction; LSE, learning self-efficacy; LI, learning initiative; SLA, self-directed learning ability; CTA, clinical thinking ability; APS, analytical and problem-solving skills; CTS, critical thinking skills.

### 3.3 Power analysis

Power analysis is used to determine the probability that a study will detect an effect if one truly exists, thereby ensuring the reliability of the study (18). According to the observed effect size for the outcomes of KS (SMD = 0.95, 95 % CI = 0.72–1.18) and PS (SMD = 1.48, 95 % CI = 1.20–1.77), the average sample size per study, and the number of included studies (*n* = 57 and *n* = 50, respectively), a precision check in GPower (two-tailed α = 0.05) was determined that the meta-analysis had sufficient power (>80%) to detect a significant effect.

### 3.4 Quality assessment

The quality of the included studies according to the Cochrane risk of bias 2 and MINORS scale is described in Table 1. Of the randomized controlled trials, 33 studies were rated as low risk, 25 as some concern, and 3 as high risk, as shown in Table 1 and Appendix 3. The majority of included studies were of high quality for overall assessment. The most common source of bias was classified as unclear due to insufficient reporting of concealment methods, insufficient information, and insufficient pre-specified analysis plan. For non-randomized controlled trials assessed using MINORS scale, the overall bias score for each article ranges from 1 to 12 for low quality, 13 to 18 for medium quality, and 19 to 24 for high quality. The quality was high in 2 studies and medium in 15 studies, as shown in Table 1 and Appendix 4. The most common source of bias was insufficient anticipated data collection information, non-concurrent controls and failing to estimate the sample size.

### 3.5 Publication bias and sensitivity analysis

**Figure 2:**
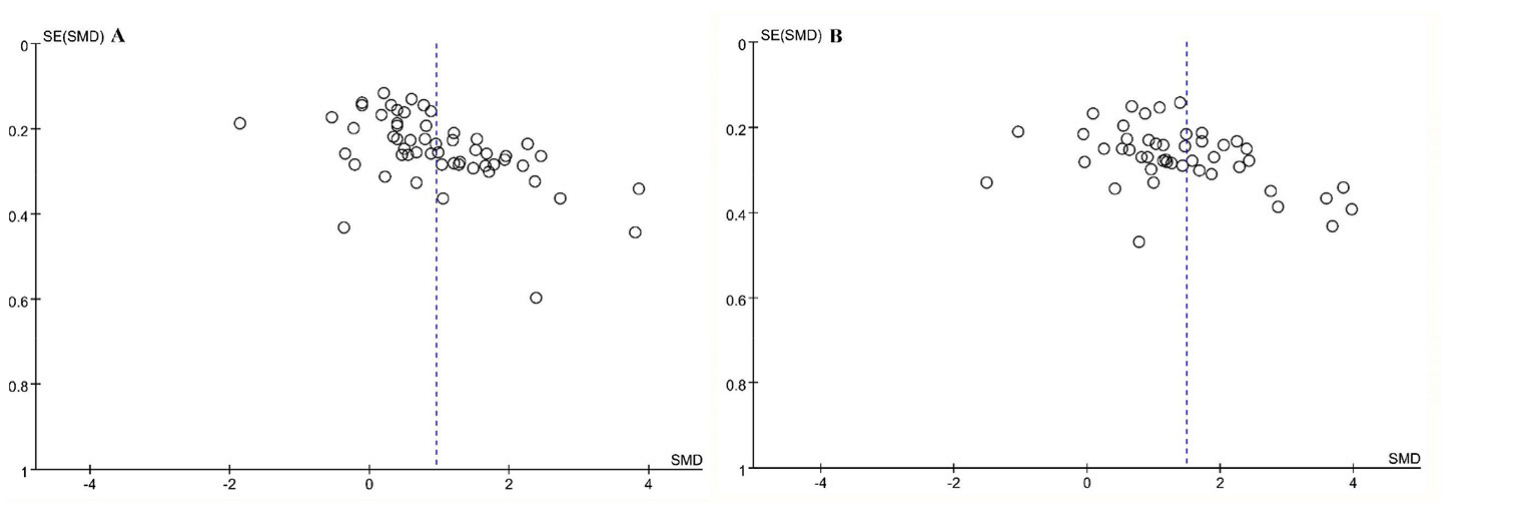
The funnel plots for the primary outcomes of KS **(A)** and PS **(B)**.

The shapes of the funnel plots for the primary outcomes of KS and PS were shown in Figure 2, while those for the secondary outcomes of TS, LSE, LI, SLA, CTA, APS, and CTS were shown in Appendix 5. The funnel plot for the secondary outcomes of TS, LSE, LI, SLA, CTA, APS and CTS was nearly symmetrical, with no evidence of publication bias (*p* > 0.05). However, the funnel plot for the primary outcomes of KS and PS showed slight asymmetry and significant evidence of publication bias (*p* < 0.05). After applying the trim-and-fill method to estimate the number of missing studies and adjust the effect size for the KS and PS, no studies requiring imputation, and the pooled effect estimate remained virtually unchanged. Subsequently, a sensitivity analysis was conducted using the one-study-removed method for the primary outcomes presented in Appendix 6. This analysis revealed that the overall meta-analytic finding is robust and not overly dependent on any single included study. Therefore, the main findings of this meta-analysis are less affected by publication bias, and the main conclusions remain robust.

### 3.6 Evaluation of the effectiveness

#### 3.6.1 Knowledge examination scores

**Figure 3:**
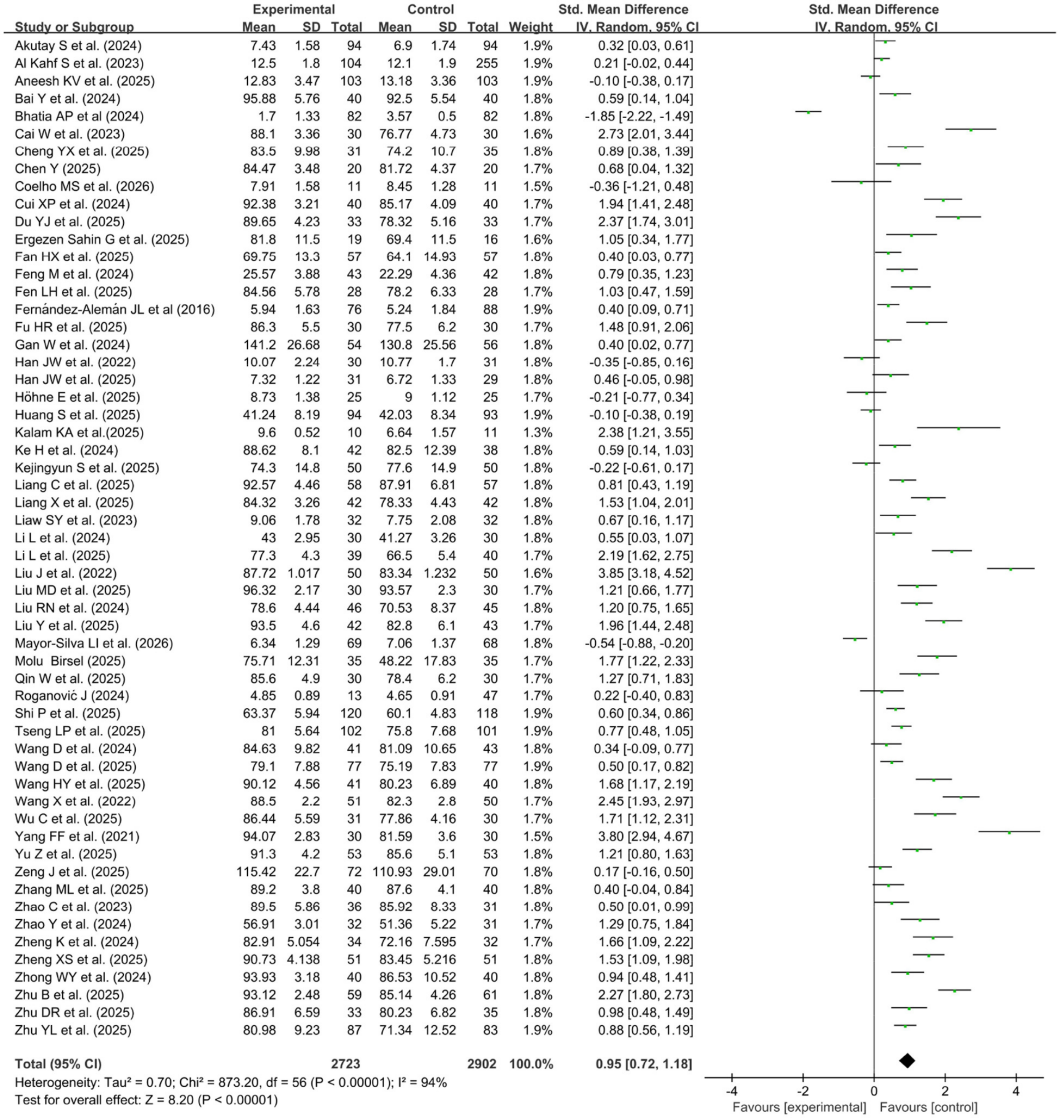
Forest plot of knowledge examination scores.

Figure 3 showed the pooled knowledge examination scores from 57 studies (19–75), which included 2,902 participants in the control group and 2,723 participants in the experimental (GAI-assisted teaching) group. A random effects model was used for the meta-analysis due to the high heterogeneity of the data (*p* < 0.00001, *I*^2^ = 94%). Compared with the control group, the pooled effect of the studies (SMD = 0.95, 95% CI: 0.72-1.18, *p* < 0.00001) showed a significant improvement in knowledge scores in the GAI-assisted teaching group.

A subgroup analysis of knowledge examination scores was performed according to geographical location, course content, knowledge level, student major and use of AI tools, as well as learning duration (Table 2). Subgroup analyses of studies reporting knowledge scores, based on subgroup classification, revealed large heterogeneity ranging from 84% to 96% among the included studies. Students in the Asian region who received GAI instruction achieved higher knowledge scores (*p* < 0.001), whereas the same method had little effect on courses in Europe and the Americas. The major and knowledge level of students, the course content and the AI tools for learning obviously influenced the comparative effectiveness of the two teaching methods in terms of theoretical knowledge achievement. Furthermore, learning with AI during the late-phase clinical stage resulted in higher knowledge scores (MSD = 1.32, *p* < 0.05), whereas the effect on pre-clinical stage learning of lower grade was low.

**Table 2:** Subgroup analysis for knowledge scores.

Subgroups classification	Data			Heterogeneity			Effects		Subgroup differences	
	Studies	Exp. (*n*)	Con. (*n*)	Chi^2^	*p*	*I* ^2^	SMD (95% CI)	*p*	*Q*-value	*p*
Geographical location	
Europe and the Americas	7	308	505	37.5	< 0.001	84	0.17 (−0.25–0.60)	0.159	21.4	**< 0.001**
Asian: China	41	1,887	1,874	485.6	< 0.001	92	1.23 (0.98–1.47)	< 0.001		
Other Asian Regions	9	528	523	188.4	< 0.001	96	0.30 (−0.34–0.93)	0.023		
Course content	
Basic medical sciences	9	533	545	286.1	< 0.001	97	0.78 (−0.02–1.58)	0.056	5.90	0.434
Internal medicine (clinical)	12	583	731	74.7	< 0.001	85	1.00 (0.68–1.32)	< 0.001		
Surgery (clinical)	11	529	522	43.5	< 0.001	77	0.81 (0.54–1.08)	< 0.001		
Medical imaging	9	343	344	170.2	< 0.001	95	1.74 (0.91–2.57)	< 0.001		
Dentistry	5	181	214	63.2	< 0.001	94	0.69 (−0.27–1.66)	0.157		
Nursing	8	364	361	119.5	< 0.001	94	0.85 (0.19–1.51)	0.012		
Other subjects	3	190	185	38.5	0.004	95	0.41 (−0.60–1.42)	0.424		
Knowledge level	
Early-phase (pre-clinical years 1–2)	7	303	311	59.7	< 0.001	90	0.61 (0.04–1.19)	0.035	7.97	**0.047**
Mid-phase (clinical transition years 3–4)	13	697	879	118.4	< 0.001	90	0.55 (0.21–0.90)	0.001		
Late-phase (clinical years & internship)	13	497	503	172.8	< 0.001	93	1.32 (0.79–1.86)	< 0.001		
Not reported	24	1,126	1,209	452.1	< 0.001	95	1.08 (0.69–1.47)	< 0.001		
Major of students	
Clinical medical major	39	1,819	1,977	456.4	< 0.001	92	1.13 (0.89–0.38)	< 0.001	4.00	0.261
Nursing major	11	622	613	167.2	< 0.001	94	0.71 (0.22–1.20)	0.005		
Dental major	6	263	296	173.7	< 0.001	97	0.26 (−0.91–1.42)	0.668		
Therapy major	1	19	16	–	–	–	1.0 (0.37–1.79)	0.003		
AI Tools	
ChatGPT	14	630	660	276.1	< 0.001	95	0.66 (0.11–1.21)	0.020	9.99	0.125
DeepSeek	3	114	113	29.7	< 0.001	93	1.49 (0.35–0.32)	0.012		
Kimi	2	72	75	10.0	0.002	90	1.60 (0.41–2.79)	0.009		
Self-developed platform	12	501	507	168.8	< 0.001	93	1.36 (0.81–1.91)	< 0.001		
Other AI tools	10	512	657	85.5	< 0.001	89	0.51 (0.11–0.91)	0.012		
Multiple AI tools	3	281	280	40.4	< 0.001	95	1.20 (0.36–2.03)	0.005		
Not reported	13	613	610	124.8	< 0.001	90	1.00 (0.61–1.40)	< 0.001		
Learning duration	
Long-term intervention	17	900	910	176.6	< 0.001	91	1.26 (0.92–1.60)	< 0.001	7.39	0.117
Medium-term intervention	7	332	477	62.2	< 0.001	90	0.70 (0.19–1.22)	0.008		
Short-term intervention	13	618	621	165.5	< 0.001	93	0.77 (0.32–1.22)	0.001		
Single-session intervention	13	576	602	336.9	< 0.001	96	0.71 (0.03–1.39)	0.042		
Not reported	7	297	292	23.9	0.001	75	1.29 (0.93–1.65)	< 0.001		

Bold indicates significance at *p* < 0.05.

#### 3.6.2 Practical examination scores

**Figure 4:**
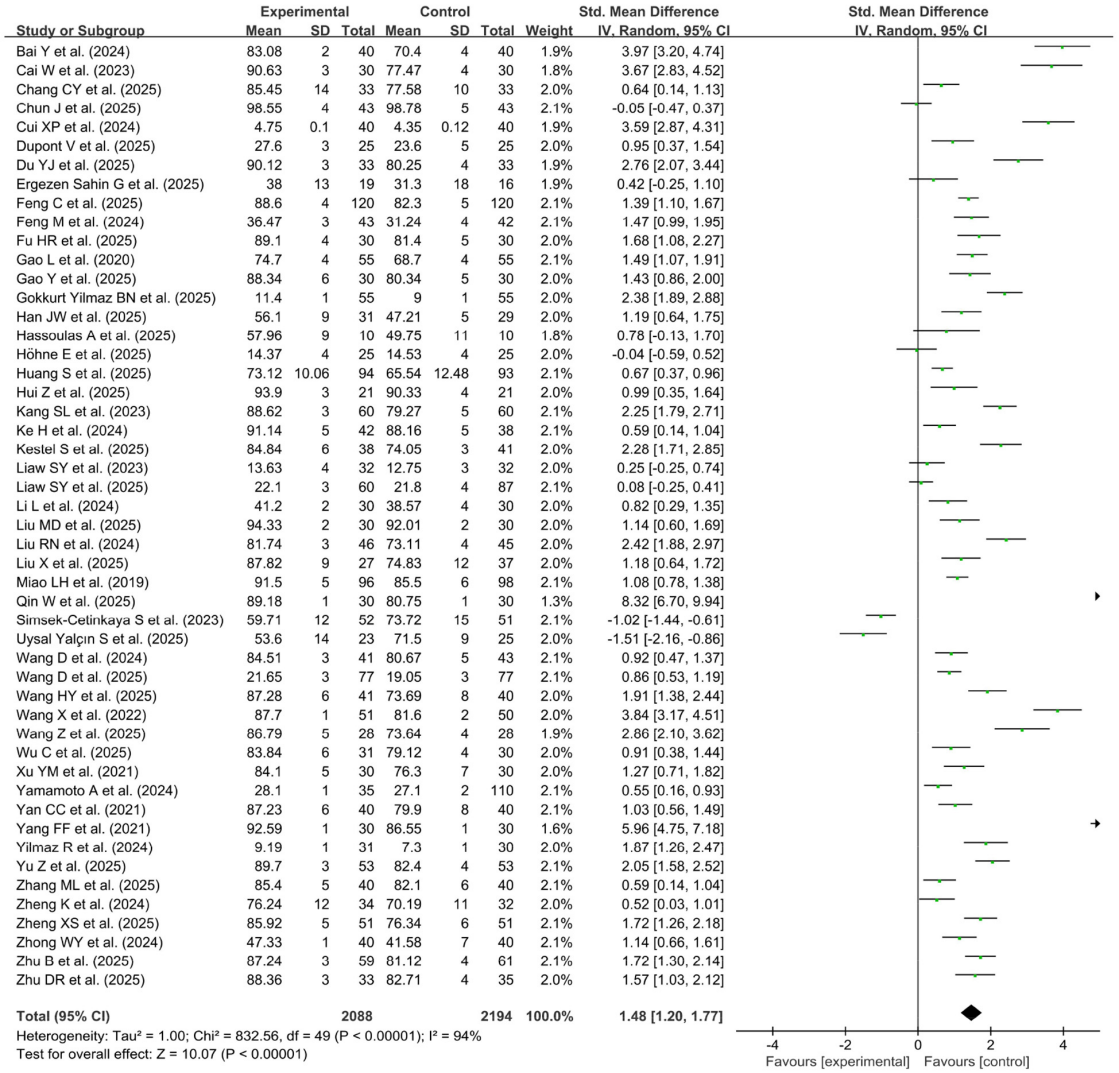
Forest plot of practical examination scores.

Figure 4 showed the pooled practical examination scores from the 50 studies (23, 24, 26, 29, 32, 34–37, 39, 40, 42, 43, 47, 48, 52, 54, 55, 60–67, 70, 72, 74, 76–96), which included 2,194 participants in the control group and 2,088 participants in the experimental group. A random effects model was used for the meta-analysis due to the high heterogeneity of the data (*p* < 0.00001, *I*^2^ = 94%). Compared with the control group, the pooled effect of the studies (SMD = 1.48, 95%CI: 1.20–1.77, *p* < 0.00001) showed a significant improving effect on experimental skills scores in the group of GAI-assisted experiment teaching group.

A subgroup analysis of practical examination scores was performed according to geographical location, course content, knowledge level, student major, use of AI tools and learning duration (Table 3). Subgroup analyses of studies reporting practical scores, based on subgroup classification, reveal large heterogeneity ranging from 75% to 98% among the included studies. Students of AI instruction in the China, Europe and Americas region achieved higher practical scores (*p* < 0.001), whereas it had little effect on courses in other Asian regions. The students' major, knowledge level, and the learning course content, the use GAI Tools for learning, and the timing of learning obviously influenced the comparative effectiveness of the two teaching methods on practical achievement. Furthermore, learning with AI during the clinical learning phase (the late phase) resulted in higher practical scores (MSD = 1.78, *p* < 0.05), whereas its effect on learning in the pre-clinical phase of lower grade was low.

**Table 3:** Subgroup analysis for practical scores.

Subgroups classification	Data			Heterogeneity			Effects		Subgroup differences	
	Studies	Exp. (*n*)	Con. (*n*)	Chi^2^	*p*	*I* ^2^	SMD (95% CI)	*p*	*Q*-value	*p*
Geographical location	
Europe and the Americas	4	91	90	21.4	< 0.001	86	0.91 (0.05–1.77)	0.039	17.1	**< 0.001**
Asian: China	35	1,576	1,582	467.5	< 0.001	93	1.88 (1.57–2.19)	< 0.001		
Other Asian Regions	11	421	522	206.3	< 0.001	95	0.45 (−0.19–1.12)	0.147		
Course content	
Basic medical sciences	3	141	153	2.3	0.313	14	1.51 (1.23–1.75)	< 0.001	12.31	0.050
Internal medicine (clinical)	8	275	274	72.8	< 0.001	90	1.66 (1.01–2.32)	< 0.001		
Surgery (clinical)	10	395	389	36.3	< 0.001	75	1.16 (0.84–1.47)	< 0.001		
Medical imaging	11	418	419	185.6	< 0.001	95	2.11 (1.38–2.83)	< 0.001		
Dentistry	3	157	156	113.0	< 0.001	98	3.84 (0.86–6.82)	0.012		
Nursing	7	307	307	155.4	< 0.001	96	1.06 (0.11–2.01)	0.029		
Other subjects	8	395	496	180.3	< 0.001	96	0.80 (0.04–1.56)	0.040		
Knowledge level	
Early-phase (pre-clinical years 1–2)	6	249	248	105.4	< 0.001	95	0.68 (−0.25–1.61)	0.151	14.74	**0.002**
Mid-phase (clinical transition years 3–4)	10	402	487	127.4	< 0.001	93	0.74 (0.18–1.30)	0.010		
Late-phase (clinical years & internship)	19	735	764	293.0	< 0.001	94	1.78 (1.29–2.27)	< 0.001		
Not reported	15	702	695	248.3	< 0.001	94	2.01 (1.18–2.55)	< 0.001		
Major of student	
Clinical medical major	34	1,357	1,428	342.7	< 0.001	90	1.58 (1.30–1.86)	< 0.001	45.97	**< 0.001**
Nursing major	11	476	502	280.8	< 0.001	96	0.83 (0.08–1.59)	0.031		
Dental major	4	212	211	130.3	< 0.001	98	3.38 (1.52–5.24)	< 0.001		
Therapy major	1	43	43	–	–	–	−0.05(−0.05–0.37)	0.814		
AI Tools	
ChatGPT	10	400	497	142.2	< 0.001	94	0.79 (0.19–1.38)	0.009	9.27	0.159
DeepSeek	3	114	113	32.0	< 0.001	94	1.75 0.50–3.01)	0.006		
Kimi	1	33	35	–	–	–	1.59 (1.05–2.14)	< 0.001		
Self-developed platform	18	816	822	291.4	< 0.001	94	1.44 (0.97–1.91)	< 0.001		
Other AI tools	6	226	226	94.6	< 0.001	95	1.83 (0.85–2.80)	< 0.001		
Multiple AI tools	2	92	94	10.6	0.001	91	1.20 (0.14–2.25)	0.027		
Not reported	10	407	407	220.5	< 0.001	96	2.22 (1.37–3.07)	< 0.001		
Learning duration	
Long-term intervention	10	413	420	126.9	< 0.001	93	1.56 (0.96–2.16)	< 0.001	1.09	0.896
Medium-term intervention	5	275	274	18.9	0.001	79	1.31 (0.88–1.74)	< 0.001		
Short-term intervention	18	717	830	376.8	< 0.001	95	1.45 (0.89–2.00)	< 0.001		
Single-session intervention	9	384	375	259.6	< 0.001	97	1.57 (0.59–2.54)	0.002		
Not reported	8	299	295	47.5	< 0.001	85	1.64 (1.14–2.13)	< 0.001		

Bold indicates significance at *p* < 0.05.

### 3.7 Secondary outcomes

#### 3.7.1 Student satisfaction for teaching

**Figure 5:**
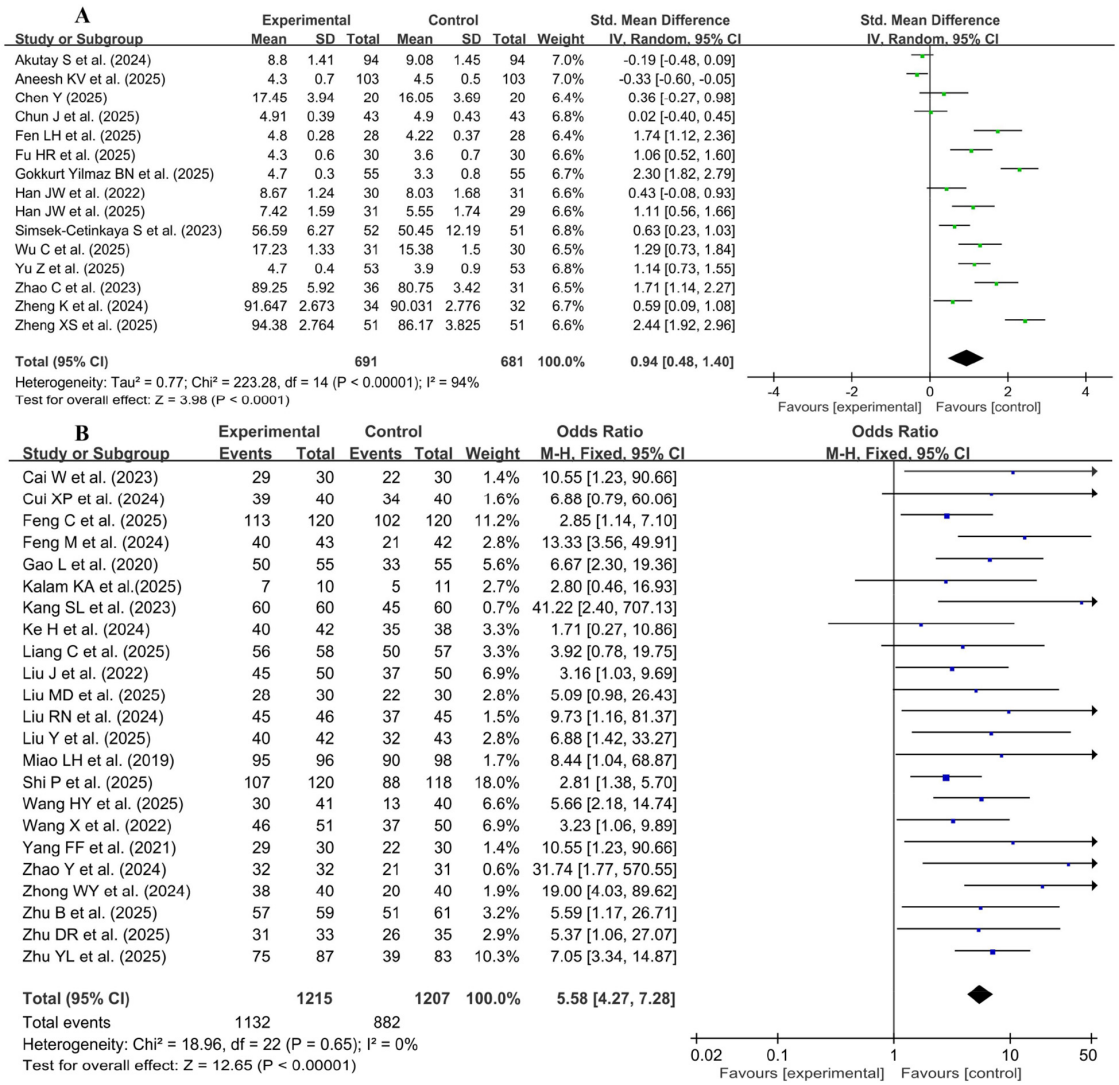
Forest plot of student satisfaction **(A)** continuous variables, **(B)** dichotomous variables.

Fifteen studies used continuous variables (20, 21, 26, 29, 37, 42, 43, 46, 54, 56, 69, 71, 77, 78, 91), and 23 studies (19, 36, 39–41, 44, 45, 50, 52, 59, 61–63, 65, 66, 70, 72–74, 87, 92, 95, 96) used dichotomous variables, which were pooled to evaluate the satisfaction for teaching in Figure 5. Due to the high heterogeneity of the data (*p* < 0.00001, *I*^2^ = 96%), a random effects model was used for the meta-analysis of continuous variables, while a fixed effects model was used for the meta-analysis of dichotomous variables due to the low heterogeneity of the data (*p* = 0.65, *I*^2^ = 0%). The meta-analysis results showed a significant improvement in teaching satisfaction in the experimental group compared with the control group (SMD = 1.52, 95% CI: 1.01–2.02, *p* < 0.00001). Furthermore, studies using dichotomous variables revealed statistically significant differences between the two groups (OR = 5.58, 95% CI: 4.27–7.28, *p* < 0.0001).

#### 3.7.2 Learning self-efficacy

**Figure 6:**
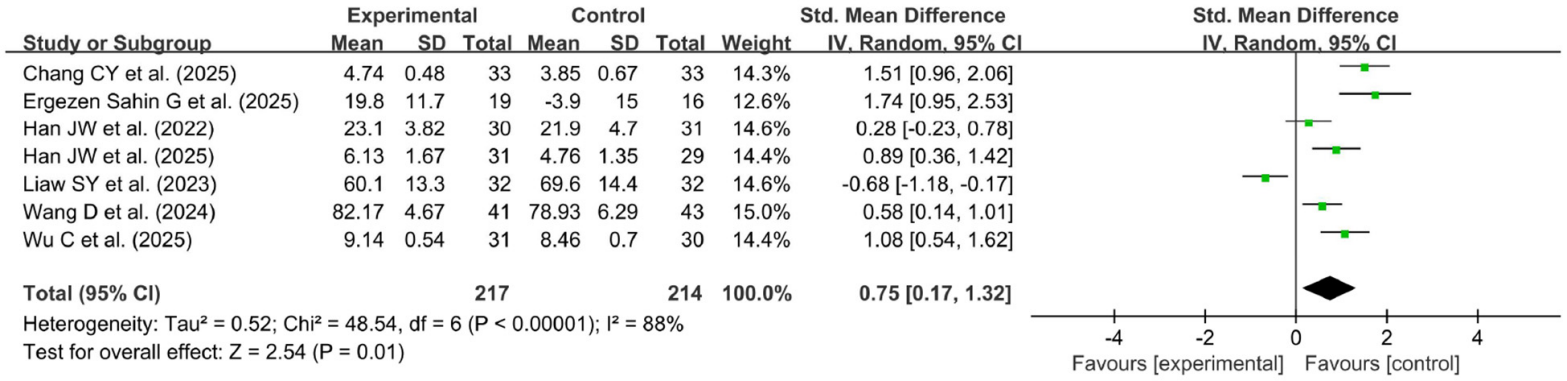
Forest plot of learning self-efficacy.

Seven studies with continuous variables were used to assess learning self-efficacy (24, 26, 29, 55, 67, 71, 76). Due to the high heterogeneity of the data (*p* < 0.00001, *I*^2^ = 88%), a random effects model was used for the meta-analysis in Figure 6. The results of the meta-analysis showed a significant improvement in learning self-efficacy in the experimental group compared with the control group (SMD = 0.75, 95% CI: 0.17–1.32, *p* < 0.00001). Furthermore, a study that used dichotomous variables revealed no statistical difference between the two groups (OR = 3.50, 95% CI: 0.92–13.32, *p* = 0.06) (81).

#### 3.7.3 Learning initiative

**Figure 7:**
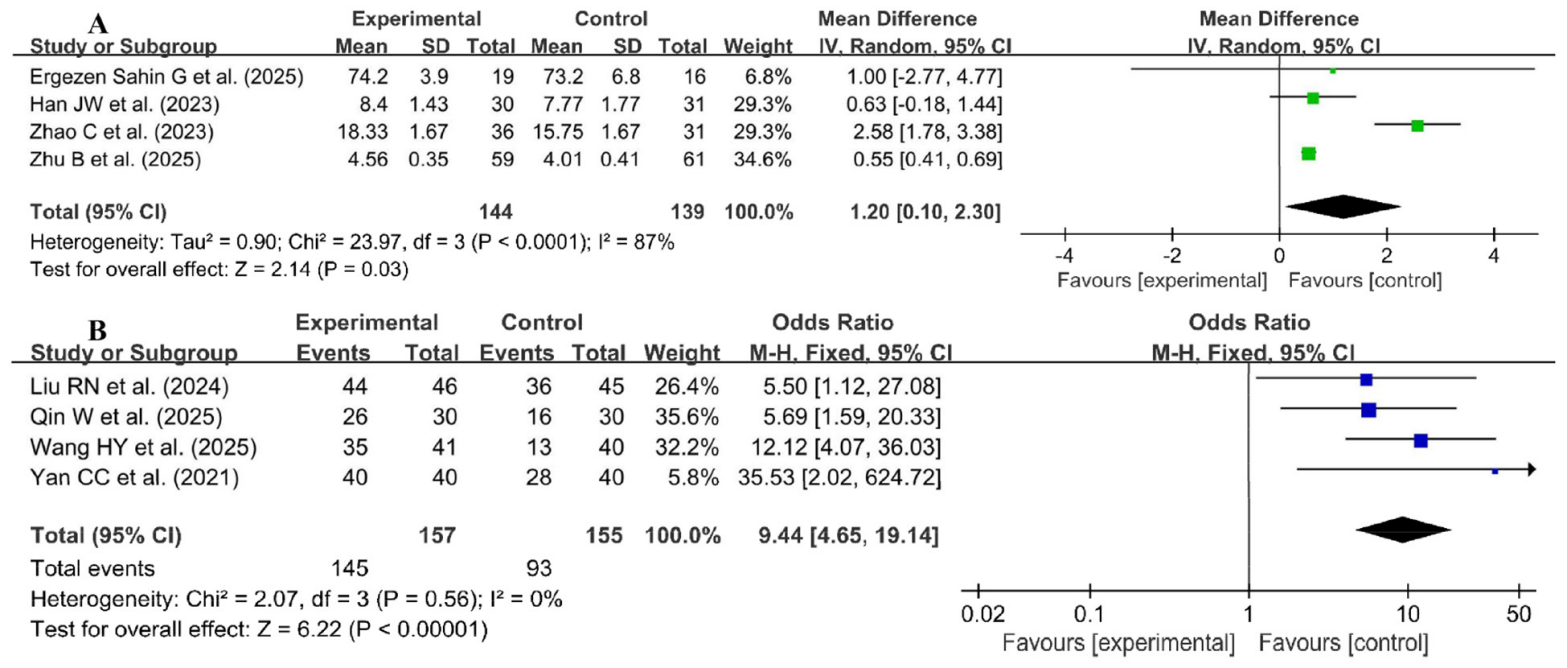
Forest plot of learning initiative **(A)** continuous variables, **(B)** dichotomous variables.

Four studies used continuous variables (24, 40, 69, 71), and four studies used dichotomous variables (35, 52, 65, 94), which were pooled to evaluate the learning initiative in Figure 7. Due to high heterogeneity for continuous variables (*p* < 0.00001, *I*^2^ = 87%), a random effects model was used for the meta-analysis. The results of the meta-analysis showed a significant improvement in learning initiative in the experimental group compared with the control group (SMD = 1.20, 95% CI: 0.10–2.30, *p* < 0.00001). Furthermore, a fixed effects model was used for the meta-analysis of the four studies with dichotomous variables (*p* = 0.56, *I*^2^ = 0%), which revealed statistically significant differences in learning initiative between the two groups (OR = 9.44, 95% CI: 4.65–19.14, *p* < 0.00001).

#### 3.7.4 Self-directed learning ability

**Figure 8:**
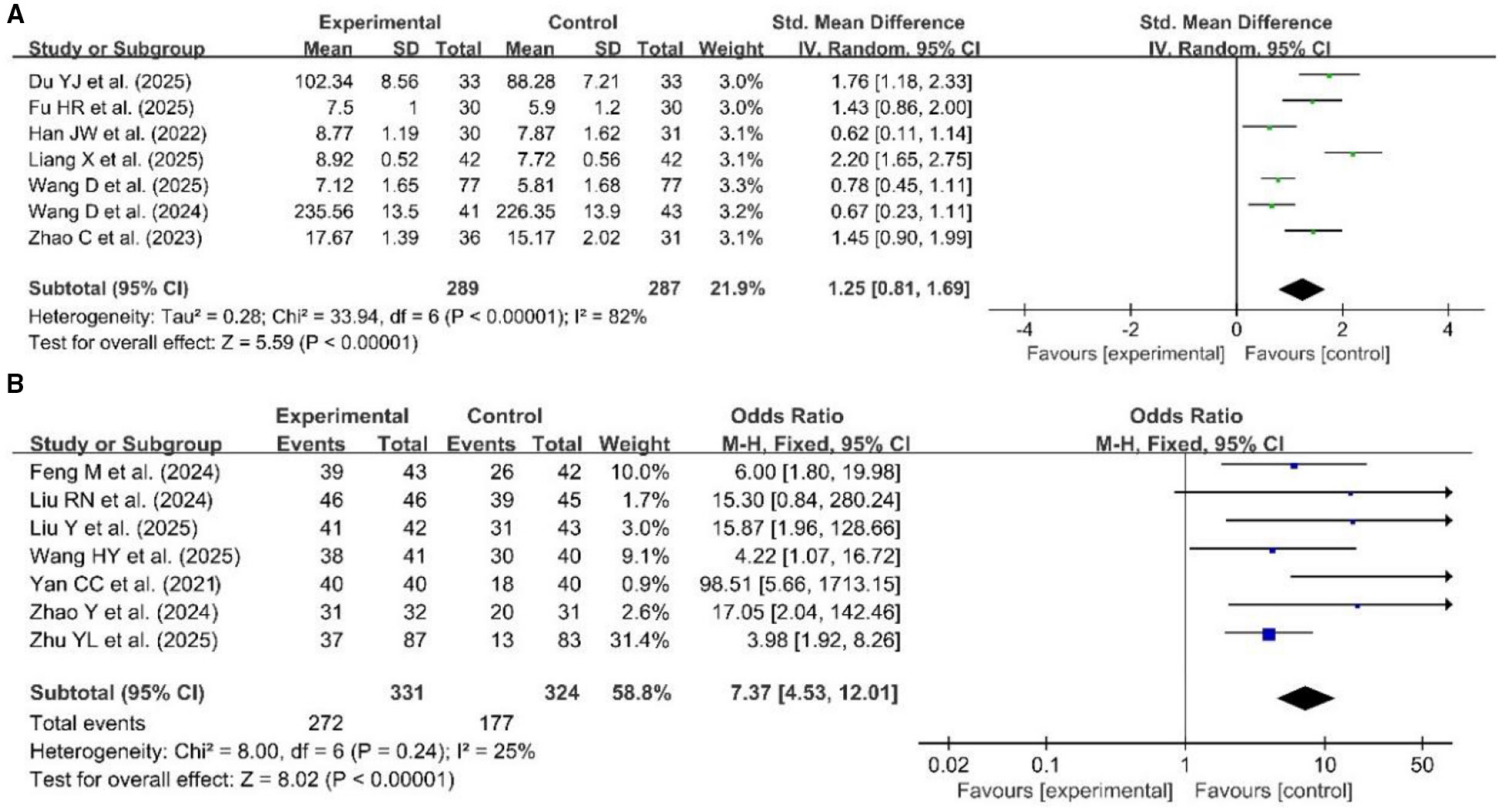
Forest plot of self-directed learning ability **(A)** continuous variables, **(B)** dichotomous variables.

Seven studies used continuous variables (34, 37, 47, 49, 55, 69, 71), and seven studies used dichotomous variables (44, 45, 52, 59, 63, 65, 94), which were pooled to evaluate the self-directed learning ability in Figure 8. Due to high heterogeneity for continuous variables (*p* < 0.00001, *I*^2^ = 82%), a random effects model was used for the meta-analysis. The results of the meta-analysis showed a significant improvement in self-directed learning ability in the experimental group compared with the control group (SMD = 1.25, 95% CI: 0.81–1.69, *p* < 0.00001). Furthermore, a fixed effects model was used for the meta-analysis of the seven studies with dichotomous variables (*p* = 0.24, *I*^2^ = 25%), which revealed statistically significant differences in self-directed learning ability between the two groups (OR = 7.37, 95% CI: 4.53–12.01, *p* < 0.00001).

#### 3.7.5 Clinical thinking ability

**Figure 9:**
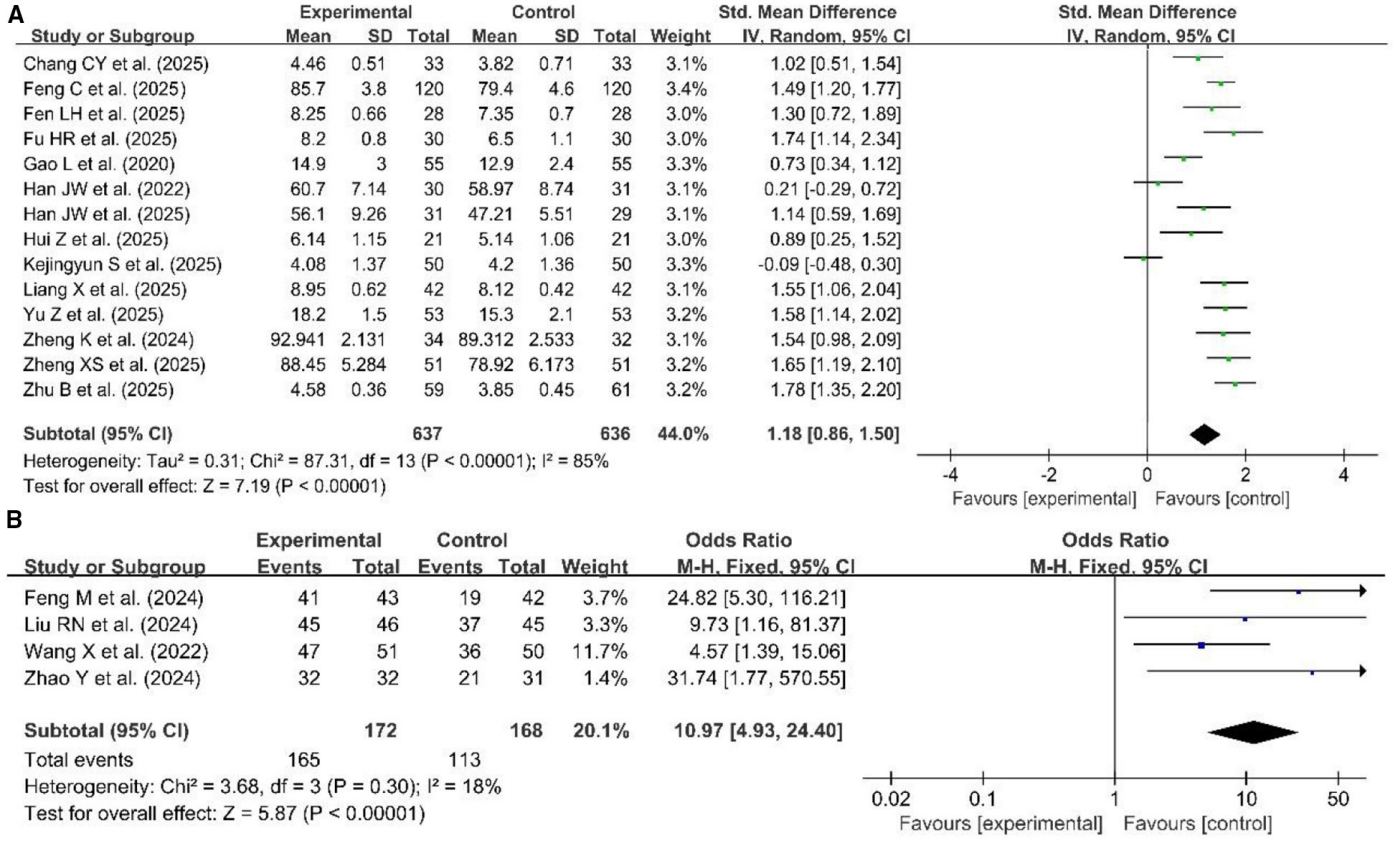
Forest plot of clinical thinking ability **(A)** continuous variables, **(B)** dichotomous variables.

Fourteen studies used continuous variables (26, 28, 37, 40, 42, 43, 46, 49, 54, 71, 76, 82, 87, 95), and four studies used dichotomous variables (59, 63, 65, 72), which were pooled to evaluate the clinical thinking ability in Figure 9. Due to high heterogeneity for continuous variables (*p* < 0.00001, *I*^2^ = 85%), a random effects model was used for the meta-analysis. The results of the meta-analysis showed a significant improvement in clinical thinking ability in the experimental group compared with the control group (SMD = 1.18, 95% CI: 0.86–1.50, *p* < 0.00001). A fixed effects model was also used for the meta-analysis of the four dichotomous variable studies (*p* = 0.30, *I*^2^ = 18%), which revealed statistically significant differences in clinical thinking ability between the two groups (OR = 10.97, 95% CI: 4.93–24.40, *p* < 0.00001).

#### 3.7.6 Analytical and problem-solving skills

**Figure 10:**
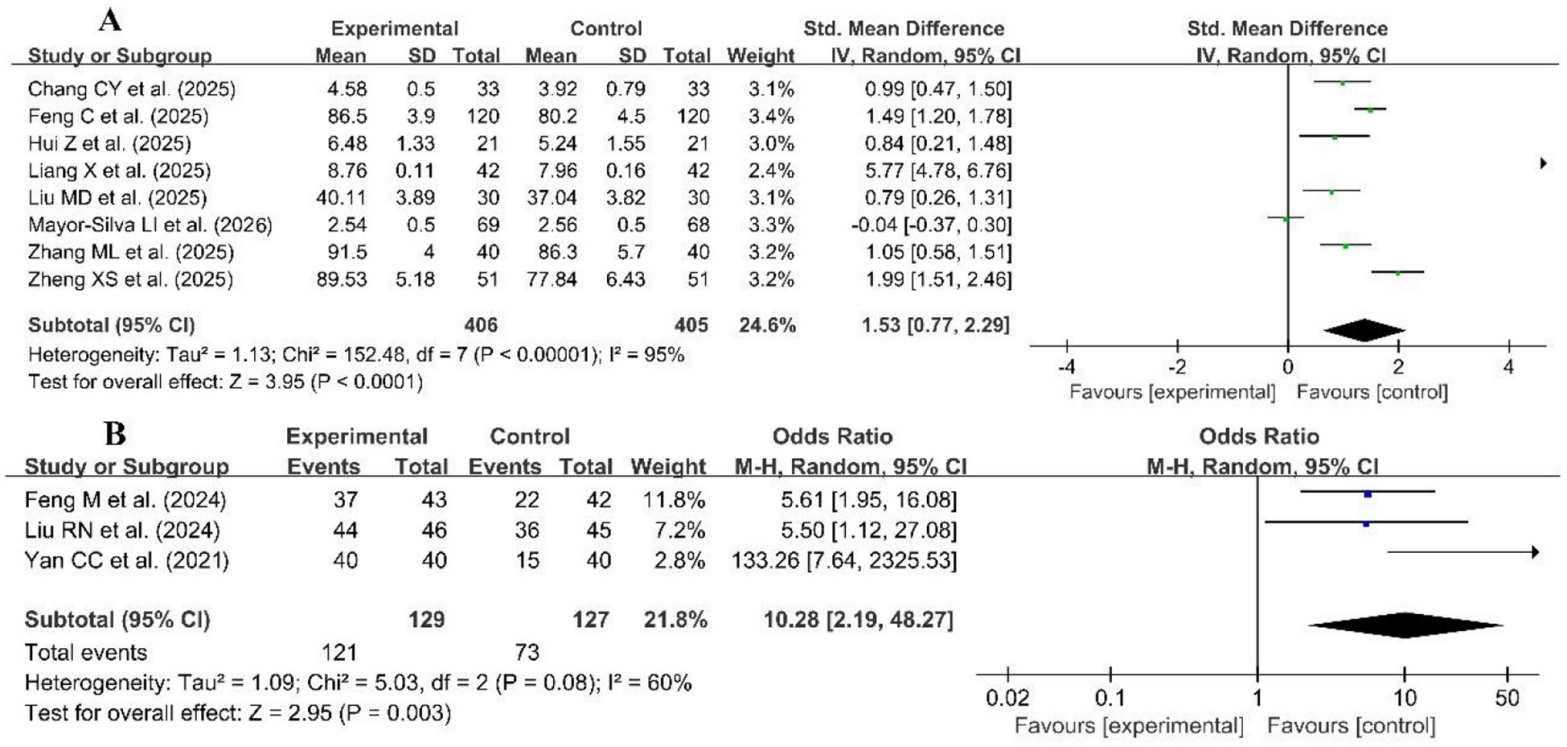
Forest plot of analytical and problem-solving skills **(A)** continuous variables, **(B)** dichotomous variables.

Eight studies used continuous variables (30, 39, 42, 48, 49, 76, 82, 87), and three studies used dichotomous variables (63, 65, 94), which were pooled to evaluate the analytical and problem-solving skills in Figure 10. Due to high heterogeneity for continuous variables (*p* < 0.00001, *I*^2^ = 95%), a random effects model was used for the meta-analysis. The results of the meta-analysis showed a significant improvement in analytical and problem-solving skills in the experimental group compared with the control group (SMD = 1.53, 95% CI: 0.77–2.29, *p* < 0.00001). A random effect was also used for the meta-analysis of the three dichotomous variable studies (*p* = 0.08, *I*^2^ = 60%), which revealed statistically significant differences in analytical and problem-solving skills between the two groups (OR = 10.28, 95% CI: 4.85–22.82, *p* < 0.00001).

#### 3.7.7 Critical thinking skills

**Figure 11:**
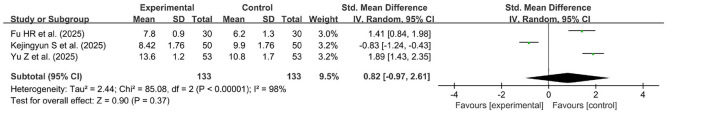
Forest plot of critical thinking skills (continuous variables).

Seven studies used continuous variables (28, 37, 43), and one study used dichotomous variables (94), which were pooled to evaluate the critical thinking skills in Figure 11. Due to high heterogeneity for continuous variables (*p* < 0.00001, *I*^2^ = 98%), a random effects model was used for the meta-analysis. The results of the meta-analysis showed no difference in critical thinking skills between the two groups (SMD = 0.82, 95% CI: −0.97–2.61, *p* = 0.37). However, only the study using dichotomous variables revealed a statistical difference between the two groups (OR = 73.47, 95%CI: 4.23–1276.95, *p* = 0.003).

## 4 Discussion

As artificial intelligence (AI) becomes increasingly popular in healthcare, medical education strategies are venturing into the realm of computer-assisted teaching powered by AI. AI-integrated medical education creates new opportunities for advanced teaching and learning experiences and improved learning outcomes (97). This meta-analysis synthesized evidence from 78 studies investigating the integration of AI in the field of medical education. A total of 3,635 medical students were included in the GAI-assisted teaching group, and 3,931 in the control group. The study showed that GAI-assisted teaching methods significantly improved medical students' outcomes, with large effect sizes observed for knowledge (SMD = 0.95), practical scores (SMD = 1.48), and the secondary outcomes (SMDs = 0.75–1.53). The ORs for all binary secondary outcomes ranged from 3.50 to 10.97. These results suggest that the GAI-assisted methodology demonstrated significant potential to enhance various dimensions of clinical, nursing and dentistry sciences education, which included improving academic performance, fostering student initiative and self-efficacy in learning, improving self-directed learning abilities, developing clinical thinking skills and analytical and problem-solving abilities, and increasing satisfaction. The results indicated that GAI-assisted pedagogy could be particularly effective in enhancing learning outcomes in medical education.

The role of AI in education can be analyzed from two key theoretical perspectives: constructivism and connectivism. The role of AI in education can be analyzed from two key theoretical perspectives: constructivism and connectivism. From a constructivist learning theory perspective, GAI functions as an intelligent scaffolding tool that facilitates active knowledge construction through personalized, interactive dialogue, enabling learners to build upon prior knowledge and develop a deeper conceptual understanding in medical contexts, as well as higher-order thinking skills, such as clinical reasoning and problem-solving (98). Concurrently, through the lens of connectivism, GAI serves as a critical node in a networked learning process, enabling students to navigate, evaluate, and synthesize diverse information sources and digital resources, which cultivated their capacity for self-directed learning and epistemic agency in a technology-rich environment (99). This synergistic support improves knowledge and practical scores directly, while also empowering learners by building their confidence in independently managing complex tasks.

Although various methods are used to assess medical students, examination results are still the most reliable indicator of their knowledge, skills and overall learning outcomes (100). Our analysis revealed significant difference in knowledge scores (*p* < 0.001) and practical scores (*p* < 0.001) between the two teaching methods. Students in the GAI-assisted teaching group achieved higher knowledge scores and practical scores than the control group (*p* < 0.001). These results demonstrate the potential effectiveness of GAI-based instruction in the teaching of medical courses. However, high between-study heterogeneity was observed irrespective of the study of knowledge scores (*I*^2^ = 94%) and practical scores (*I*^2^ = 94%). This heterogeneity is likely the result of the diversity of methods used in GAI-assisted pedagogy, and partly because there is not yet a universally accepted pedagogical implementation of the framework, resulting in uneven adoption across institutions. GAI has the potential to support personalized teaching and accommodate diverse learning styles, resulting in different learning outcomes in medical education (98). Furthermore, subgroup analysis indicated that there were significant differences in knowledge and practice scores among subgroups based on geographic location, knowledge level, and students' majors, which may also be one of the factors contributing to heterogeneity.

The included studies covered a variety of geographical locations and methodological designs, involving five developing countries and nine developed countries. Of these 78 studies, 51 (65.4%) were predominantly represented by institutions in mainland China. The adoption of AI varies significantly across different countries due to differences in regulatory frameworks, the availability of resources, and cultural attitudes toward technology. A multicenter study conducted in 48 countries reveals that the integration of AI education within medical curricula varies significantly across different regions, particularly between continents and the global north and south, which may reflect differing national AI policies, educational strategies, and macroeconomic factors (5). In China, the Ministry of Education issued the artificial intelligence innovation action plan for higher education institutions, which accelerated innovation and the application of artificial intelligence in education and promoted GAI-enabled innovation in medical education in 2018 (101). Over the next 8 years, many medical educators at China's medical universities explored the potential of GAI-empowered medical education, and then achieving positive results.

Previously published meta-analyses included studies from participants across orthodontic and clinical medical disciplines and academic levels, which limited the generalizability of their results to the field of medical education (13, 15). These analyses also demonstrated that the GAI-based teaching methods have not improved theoretical scores among medical students (15). In this study, we focused exclusively on undergraduate education in medical fields, including clinical, dentistry and nursing disciplines. The GAI-assisted teaching strategies have significantly improved students‘ knowledge and practical skills, as well as teaching satisfaction, across basic medical, clinical medical (e.g., internal medicine, surgery, medical imaging) and nursing curriculums. This improvement suggests that GAI-assisted teaching helps students to understand and apply knowledge more effectively in the classroom, ultimately improving their academic performance. Compared with the traditional teaching methods used in the control group, most of the reviewed studies indicated that students favored GAI-assisted teaching and reported higher satisfaction levels. These findings suggest that GAI-assisted teaching improves academic performance and boosts students' overall engagement, initiative, and self-efficacy when learning the subject. In the area of skill assessment, it also enhances clinical competence, including self-directed learning abilities, clinical thinking skills, and analytical and problem-solving abilities.

Although GAI currently functions primarily as a complementary educational tool in medical education (102), using the GAI-assisted teaching strategy has a positive effect on academic performance and competence. The constructivist model of learning posits that learning is an active process whereby individuals construct knowledge by linking new information to their existing knowledge and experiences (98). Connectivism emphasizes that knowledge is distributed across networks, and that learning involves navigating these connections (99). Firstly, GAI provides medical students with rapid access to accurate, up-to-date information on medical topics, enabling them to quickly identify key insights or information on specific subjects (103). Secondly, an online GAI communication platform could create a more engaging, intelligent, and approachable learning environment (104). Students who received GAI-assisted teaching were more willing to actively participate in the learning process than to passively receive information, which could enhance their engagement and initiative when learning the subject. Thirdly, traditional medical education assessments often take a long time to grade. In contrast, the online GAI platform transcends temporal and spatial constraints, which offers students immediate personalized feedback across various subjects and stages of learning (105), enabling them to make timely improvements and enhancing their learning efficiency and self-efficacy. Fourthly, when combined with lecture-based learning, problem-based learning, case-based learning, group discussions, flipped classrooms and remote learning environments, the online GAI platform presents transformative learning opportunities, partially offsetting the shortcomings of traditional teaching and ultimately enhancing the overall medical education experience (106). Finally, the GAI platform assists medical students to assess their performance during and after their courses, offer the guidance on effective learning strategies, case analyses and simulated consultations, which enhances clinical reasoning and practical skills, and fosters deeper inquiry (107).

This meta-analysis has several limitations that merit careful consideration. These are primarily related to geographic concentration, publication bias, and substantial heterogeneity. Firstly, although our systematic review examined international studies on GAI-assisted teaching in clinical dentistry and nursing, 51 of the included studies were conducted in China. This geographical concentration raises questions about the wider adoption of GAI-assisted teaching in clinical dentistry and nursing education around the world. Secondly, although our search strategy covered both English- and Chinese-language databases, the fact that many GAI-assisted teaching studies are published in Chinese contributes to language and publication biases that are common in medical education research. Thirdly, our inclusion criteria required studies to meet specific methodological standards, potentially excluding lower-quality international studies. Fourthly, there is a potential for publication bias in this study, as indicated by slight asymmetry in the funnel plot and significant evidence of publication bias in Egger's test, which could be due to the selective reporting of smaller studies with favorable outcomes. Finally, despite conducting extensive subgroup analyses that resulted in reduced heterogeneity, the remaining substantial heterogeneity must be acknowledged. Differences in study design, sample size and teaching context could explain the variation in the observed effects on the overall impact of GAI-assisted teaching. Despite these limitations, the findings of our review have broader implications for education in the field of global medicine. These limitations should be addressed in future research through the conduct of large-scale, well-designed studies that employ standardized protocols.

## 5 Conclusions

This meta-analysis included 61 RCTs and 17 observational non-randomized controlled trial with a total of 7655 students, comprising 3,931 participants in the control groups and 3,635 participants in the GAI-assisted intervention groups. The meta-analysis provided positive evidence of the effectiveness of GAI-assisted teaching methodologies in global clinical medicine, dentistry and nursing education. The findings demonstrated that the GAI-assisted teaching strategy is more effective than traditional methods in enhancing academic performance, improving learning satisfaction, initiative and self-efficacy in learning, and developing clinical competence, including self-directed learning abilities, clinical thinking skills, and analytical and problem-solving abilities, in medical education. The GAI-assisted teaching strategy appears to be more effective than other teaching methods, and using this pedagogy could be one of the best ways to improve medical education. Subgroup analyses indicated that the variables such as geographic location, level of knowledge, and students' majors had a significant impact on academic performance. In future, policymakers and education administrator should consider integrating artificial intelligence into teacher training and medical curriculum design to improve learning outcomes. Particularly, strategies for integrating AI into the medical curriculum should be tailored to the knowledge level and subject specialism of specific groups of learners.

## Data Availability

The original contributions presented in the study are included in the article/Supplementary material, further inquiries can be directed to the corresponding author.
