# Supplementary material for: The effectiveness of GAI-assisted teaching methods in medical education: a systematic review and meta-analysis
Source: Front Public Health. 2026 Apr 22;14:1813108. doi: 10.3389/fpubh.2026.1813108 (PMC13143992; doi:10.3389/fpubh.2026.1813108)
Supplement: Supplementary file 2 [file Data_Sheet_1.pdf]

# Supplementary material

The effectiveness of GAI-assisted teaching methods in medical education: a systematic review and meta-analysis

| | |
|---|---|
| **Appendix 1** | PRISMA checklist |
| **Appendix 1** | literature search strategy<br>(PubMed, Web of Science, Embase, Cochrane Library, CNKI，CSTJ, Wan Fang Database and CBM) |
| **Appendix 3** | The quality assessment of each study (A) and total studies (B) for RCT study with RoB2 tool (n=61) |
| **Appendix 4** | The quality assessment of each study for non-RCT with MINORS scale (n=17) |
| **Appendix 5** | The shapes of the funnel plots for the TS, LSE, LI, SLA, CTA, APS, and CTS<br>Figure 5.1 The shapes of the funnel plots for teaching satisfaction (TS) of continuous variables<br>Figure 5.2 The shapes of the funnel plots for teaching satisfaction (TS) of dichotomous variables<br>Figure 5.3 The shapes of the funnel plots for learning self-efficacy (LSE) of continuous variables<br>Figure 5.4 The shapes of the funnel plots for critical thinking skills (CTS) of continuous variables<br>Figure 5.5 The shapes of the funnel plots for learning initiative (LI) of continuous variables<br>Figure 5.6 The shapes of the funnel plots for learning initiative (LI) of dichotomous variables<br>Figure 5.7 The shapes of the funnel plots for self-directed learning ability (SLA) of continuous variables<br>Figure 5.8 The shapes of the funnel plots for self-directed learning ability (SLA) of dichotomous variables<br>Figure 5.9 The shapes of the funnel plots for clinical thinking ability (CTA) of continuous variables<br>Figure 5.10 The shapes of the funnel plots for clinical thinking ability (CTA) of dichotomous variables<br>Figure 5.11 The shapes of the funnel plots for analytical and problem-solving skills (APS) of continuous variables<br>Figure 5.12 The shapes of the funnel plots for analytical and problem-solving skills (APS) of dichotomous variables |
| **Appendix 6** | The sensitivity analysis using the one-study-removed method for primary outcomes<br>Table 6.1 The sensitivity analysis for the outcome of knowledge examination scores<br>Table 6.2 The sensitivity analysis for the outcome of practical examination scores |

# Appendix 1 PRISMA checklist

| Section/topic | Item No | Checklist item | Reported on page No |
|---|---|---|---|
| **Title** | | | |
| Title | 1 | Identify the report as a systematic review, meta-analysis, or both | 1 |
| **Abstract** | | | |
| Structured summary | 2 | Provide a structured summary including, as applicable, background, objectives, data sources, study eligibility criteria, participants, interventions, study appraisal and synthesis methods, results, limitations, conclusions and implications of key findings, systematic review registration number | 2 |
| **Introduction** | | | |
| Rationale | 3 | Describe the rationale for the review in the context of what is already known | Introduction |
| Objectives | 4 | Provide an explicit statement of questions being addressed with reference to participants, interventions, comparisons, outcomes, and study design (PICOS) | Introduction |
| **Methods** | | | |
| Protocol and registration | 5 | Indicate if a review protocol exists, if and where it can be accessed (such as web address), and, if available, provide registration information including registration number | No |
| Eligibility criteria | 6 | Specify study characteristics (such as PICOS, length of follow-up) and report characteristics (such as years considered, language, publication status) used as criteria for eligibility, giving rationale | Methods |
| Information sources | 7 | Describe all information sources (such as databases with dates of coverage, contact with study authors to identify additional studies) in the search and date last searched | Methods |
| Search | 8 | Present full electronic search strategy for at least one database, including any limits used, such that it could be repeated | Appendix 2 |
| Study selection | 9 | State the process for selecting studies (that is, screening, eligibility, included in systematic review, and, if applicable, included in the meta-analysis) | Methods |
| Data collection process | 10 | Describe method of data extraction from reports (such as piloted forms, independently, in duplicate) and any processes for obtaining and confirming data from investigators | Methods |
| Data items | 11 | List and define all variables for which data were sought (such as PICOS, funding sources) and any assumptions and simplifications made | Methods |
| Risk of bias in individual studies | 12 | Describe methods used for assessing risk of bias of individual studies (including specification of whether this was done at the study or outcome level), and how this information is to be used in any data synthesis | Methods |
| Summary measures | 13 | State the principal summary measures (such as risk ratio, difference in means). | Methods |
| Synthesis of results | 14 | Describe the methods of handling data and combining results of studies, if done, including measures of consistency (such as $I^2$ statistic) for each meta-analysis | Methods |
| Risk of bias across studies | 15 | Specify any assessment of risk of bias that may affect the cumulative evidence (such as publication bias, selective reporting within studies) | Methods |
| Additional analyses | 16 | Describe methods of additional analyses (such as sensitivity or subgroup analyses, meta-regression), if done, indicating which were pre-specified | Methods |
| **Results** | | | |
| Study selection | 17 | Give numbers of studies screened, assessed for eligibility, and included in the review, with reasons for exclusions at each stage, ideally with a flow diagram | Figure 1 |
| Study | 18 | For each study, present characteristics for which data were extracted (such as study size, PICOS, follow-up | Table 1 |

| Section/topic | Item No | Checklist item | Reported on page No |
|---|---|---|---|
| characteristics | | period) and provide the citations | |
| Risk of bias within studies | 19 | Present data on risk of bias of each study and, if available, any outcome-level assessment (see item 12). | Table 1, Appendix 3/4 |
| Results of individual studies | 20 | For all outcomes considered (benefits or harms), present for each study (a) simple summary data for each intervention group and (b) effect estimates and confidence intervals, ideally with a forest plot | Figure 3-11 |
| Synthesis of results | 21 | Present results of each meta-analysis done, including confidence intervals and measures of consistency | Figure 3-11 |
| Risk of bias across studies | 22 | Present results of any assessment of risk of bias across studies (see item 15) | Figure 2, Appendix 5 |
| Additional analysis | 23 | Give results of additional analyses, if done (such as sensitivity or subgroup analyses, meta-regression) (see item 16) | Appendix 6 |
| **Discussion** | | | |
| Summary of evidence | 24 | Summarise the main findings including the strength of evidence for each main outcome; consider their relevance to key groups (such as health care providers, users, and policy makers) | Discussion |
| Limitations | 25 | Discuss limitations at study and outcome level (such as risk of bias), and at review level (such as incomplete retrieval of identified research, reporting bias) | Discussion |
| Conclusions | 26 | Provide a general interpretation of the results in the context of other evidence, and implications for future research | Discussion |
| **Funding** | | | |
| Funding | 27 | Describe sources of funding for the systematic review and other support (such as supply of data) and role of funders for the systematic review | Page 36 |

## Appendix 2 literature search strategy

**PubMed Search Strategy**

#1 "Artificial Intelligence"[Mesh] OR "Generative Artificial Intelligence"[Mesh] OR "Generative Artificial Intelligence"[Title/Abstract] OR "Generative AI"[Title/Abstract] OR "Artificial Intelligence"[Title/Abstract] OR "Chatbot*"[Title/Abstract] OR "Large Language Model"[Title/Abstract] OR "Generative Language Model"[Title/Abstract] OR "ChatGPT"[Title/Abstract] OR "DeepSeek"[Title/Abstract]

#2 "Medical Education"[Mesh] OR "Medical Education"[Title/Abstract] OR "Teaching Method*"[Title/Abstract] OR "Teaching"[Title/Abstract] OR "Medical Learning"[Title/Abstract] OR "Learning"[Title/Abstract]

#3 "Students"[Mesh] OR "Student*"[Title/Abstract]

#4 "Randomized controlled trial"[Publication Type] OR "Controlled Trial"[PT] OR "Control*"[All Fields] OR "Trial*"[All Fields]

(#1 AND #2 AND #3 AND #4) AND (("2005/01/01"[Date - Publication] : "2025/11/14"[Date - Publication])) = 488

**Web of Science Search Strategy**

#1 TS=("Generative Artificial Intelligence" OR "Generative AI" OR "Artificial Intelligence Chatbot*" OR "AI Chatbot*" OR "Large Language Model*" OR "Generative Language Model*" OR "ChatGPT" OR "DeepSeek")

#2 TS=("Medical Education" OR "Teaching" OR "Teaching Method*" OR "Medical Learning" OR "Learning")

#3 TS=("Student" OR "Student*")

#4 TS=("Randomized controlled trial*" OR "Controlled trial*" OR "Trial*" OR "Control*")

#5 #1 AND #2 AND #3 AND #4 AND Publication Date=(2005-01-01 to 2025-11-14) =359

**Elsevier ScienceDirect Search Strategy**

#1 "Artificial Intelligence" OR "Generative Artificial Intelligence" OR "Generative AI" OR "Artificial Intelligence" OR "Chatbot" OR "Large Language Model" OR "Generative Language Model" OR "ChatGPT" OR "DeepSeek"

#2 "Medical Education" OR "Teaching Method" OR "Teaching" OR "Medical Learning" OR "Learning"

#3 "Student" OR "Students"

#4 "Randomized controlled trial" OR "Controlled Trial" OR "Control" OR "Trial"

Title, abstract, keywords: ("Artificial Intelligence" OR "ChatGPT" OR "DeepSeek") AND ("Education" OR "Teaching" OR "Learning") AND ("Student") AND ("Control" OR "Trial") AND Publication Date=(2005-01-01 to 2025-11-14) =214

**Embase Search Strategy**

#1 'Generative Artificial Intelligence'/exp OR 'Generative AI'/exp OR 'Artificial Intelligence Chatbot'/exp OR 'AI Chatbot'/exp OR 'Large Language Model'/exp OR 'Generative Language Model':ti,ab OR 'ChatGPT':ti,ab OR 'DeepSeek':ti,ab

#2 'Medical Education'/exp OR 'Teaching'/exp OR 'Teaching Method*':ti,ab OR 'Learning'/exp OR ' Medical Learning':ti,ab

#3 'Student'/exp OR 'Student*':ti,ab

#4 'Randomized controlled trial'/exp OR 'Controlled trial'/exp OR 'Control*':ti,ab OR 'Trial*':ti,ab

#5 #1 AND #2 AND #3 AND #4 AND [01-01-2005 to 12-11-2025]/pd =176

**Cochrane Library Search Strategy**

#1 MeSH descriptor: [Artificial Intelligence] explode all trees

#2 ("Generative Artificial Intelligence" OR "Generative AI" OR "Artificial Intelligence Chatbot" OR "AI Chatbot" OR "Large Language Model" OR "Generative Language Model" OR "ChatGPT" OR "DeepSeek"):ti,ab,kw

#3 MeSH descriptor: [Education, Medical] explode all trees

#4 ("Medical Education" OR "Teaching Method" OR "Education" OR "Teaching" OR "Medical Learning" OR "Learning"):ti,ab,kw

#5 ("student" OR "students"):ti,ab,kw

#6 (#1 OR #2) AND (#3 OR #4) AND #5 with publication 2005 to 2025 = 176

CNKI Data

#1：主题：医学教学 ＋ 医学教育 ＋ 医学课程 ＋ 临床实践 ＋ 医学实践 ＋ 医学专业 ＋ 学生教育 ＋ 临床医学 ＋ 预防医学 ＋ 口腔医学 ＋ 护理学

#2：主题：医学生 ＋ 大学生 ＋ 本科生 ＋ 专科生

#3：主题：人工智能 ＋ 生成式人工智能 + AI + 机器人 ＋ 数智化 ＋ 深度学习 ＋ 语言模型 ＋ 自然语言处理 + ChatGPT + DeepSeek

#1 AND #2 AND #3 (2025-01-01-2025-10-24) = 1503

**Cqvip Data**

#1：题名或关键词；医学教学 + 医学教育 + 医学课程 + 临床实践 + 医学实践 + 学生教育 + 临床医学 + 预防医学 + 口腔医学 + 护理学

#2：题名或关键词；医学生 + 大学生 + 本科生 + 专科生

#3：题名或关键词；人工智能 + 生成式人工智能 + AI + 机器人 + 数智化 + 深度学习 + 语言模型 + 自然语言处理 + ChatGPT + DeepSeek

#1 AND #2 AND #3 (2025-01-01-2025-10-24) = 589

**Wanfang Data**

#1:题名或关键词:医学教学 OR 医学教育 OR 医学课程 OR 临床实践 OR 医学实践 OR 学生教育 OR 临床医学 OR 预防医学 OR 口腔医学 OR 护理学

#2：题名或关键词：医学生 OR 大学生 OR 本科生 OR 专科生

#3：题名或关键词：人工智能 OR 生成式人工智能 OR AI OR 机器人 OR 数智化 OR 深度学习 OR 语言模型 OR 自然语言处理 OR ChatGPT OR DeepSeek

#1 AND #2 AND #3 (2025-01-01-2025-10-24) = 1183

**CBM Data**

#1：[常用字段:智能]：医学教学，医学教育，医学课程，临床实践，医学实践，医学专业，学生教育，临床医学，预防医学，口腔医学，护理学，

#2：[常用字段:智能]：医学生，大学生，本科生，专科生

#3：[常用字段:智能]：人工智能，生成式人工智能，AI，机器人，数智化，深度学习，大语言模型，自然语言处理，ChatGPT，DeepSeek

#1 AND #2 AND #3 (2025-01-01-2025-10-24) = 976

**Appendix 3 The quality assessment of each study (A) and total studies (B) for RCT study with RoB2 tool (n=61)**

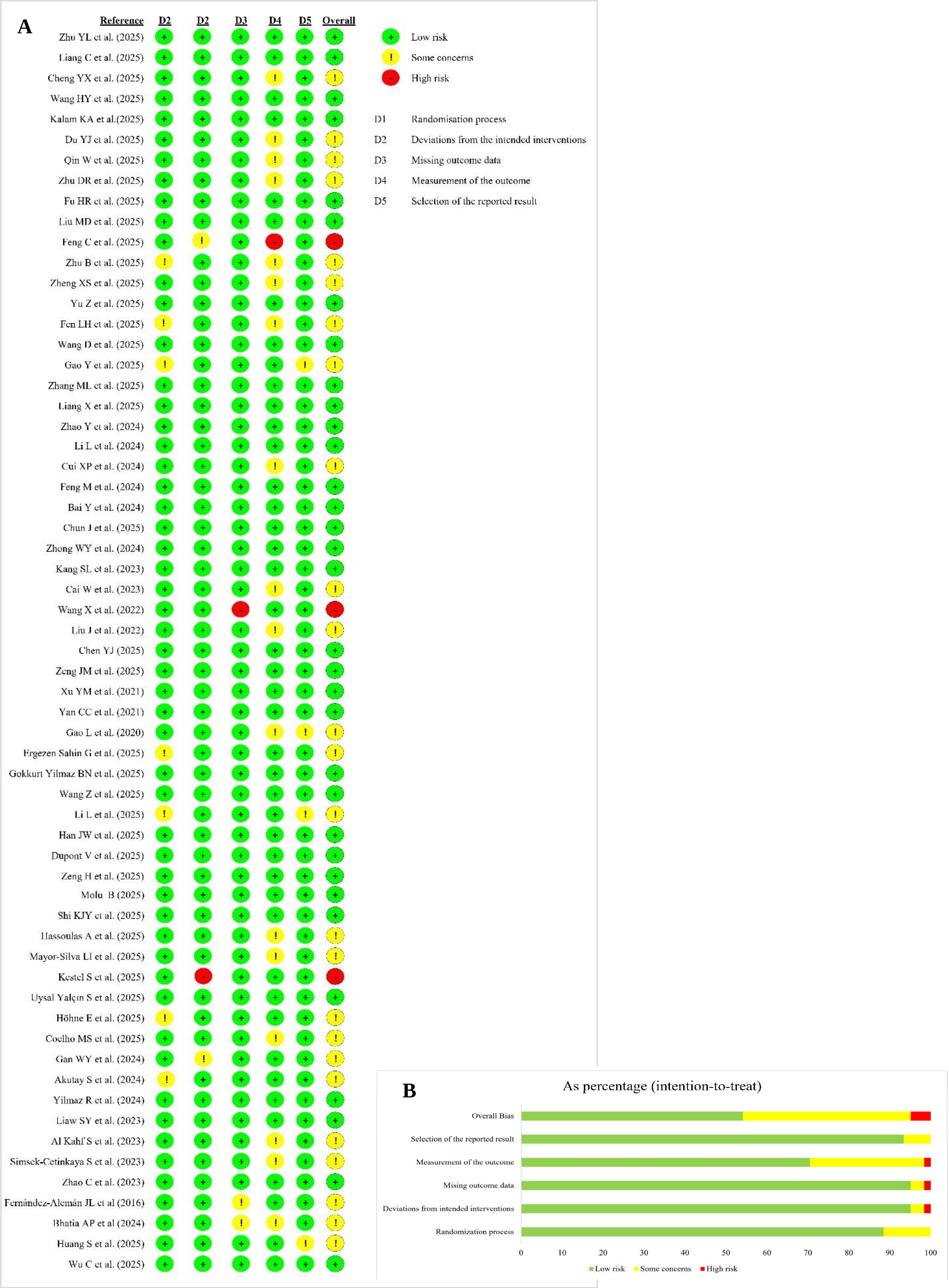

**Appendix 4 The quality assessment of each study for non-RCT with MINORS scale (n=17)**

**Evaluation index**:1.A clearly stated aim; 2.Inclusion of consecutive patients; 3.Prospective collection of data; 4. Endpoints appropriate to the aim of the study; 5. Unbiased assessment of the study endpoint; 6. Follow-up period appropriate to the aim of the study; 7. Loss to follow up less than 5%; 8. Prospective calculation of the study size; 9. An adequate control group; 10. Contemporary groups; 11. Baseline equivalence of groups; 12. Adequate statistical analyses.

| **Author (year)** | **Evaluation index** | | | | | | | | | | | | **Scores** | **Assessment** |
|---|---|---|---|---|---|---|---|---|---|---|---|---|---|---|
| | 1 | 2 | 3 | 4 | 5 | 6 | 7 | 8 | 9 | 10 | 11 | 12 | | |
| Aneesh KV et al. (2025) | 2 | 1 | 2 | 2 | 1 | 2 | 2 | 0 | 1 | 2 | 2 | 2 | 19 | High quality |
| Chang CY et al. (2025) | 2 | 1 | 1 | 2 | 1 | 2 | 2 | 0 | 1 | 2 | 2 | 2 | 18 | Medium quality |
| Liu X et al. (2025) | 2 | 1 | 1 | 2 | 1 | 2 | 2 | 0 | 1 | 2 | 2 | 2 | 18 | Medium quality |
| Liaw SY et al. (2025) | 2 | 1 | 1 | 2 | 1 | 2 | 2 | 0 | 1 | 2 | 2 | 2 | 18 | Medium quality |
| Tseng LP et al. (2025) | 2 | 1 | 1 | 2 | 1 | 2 | 2 | 0 | 1 | 2 | 2 | 2 | 18 | Medium quality |
| Yamamoto A et al. (2024) | 2 | 1 | 1 | 2 | 1 | 2 | 2 | 0 | 1 | 2 | 2 | 2 | 18 | Medium quality |
| Zheng K et al. (2024) | 2 | 1 | 1 | 2 | 1 | 2 | 2 | 0 | 1 | 2 | 2 | 2 | 18 | Medium quality |
| Wang DX et al. (2024) | 2 | 1 | 1 | 2 | 1 | 2 | 2 | 0 | 1 | 2 | 2 | 2 | 18 | Medium quality |
| Roganović J (2024) | 2 | 1 | 1 | 2 | 1 | 2 | 2 | 0 | 1 | 2 | 2 | 2 | 18 | Medium quality |
| Han JW et al. (2023) | 2 | 2 | 2 | 2 | 1 | 2 | 2 | 2 | 2 | 2 | 2 | 2 | 23 | High quality |
| Fan HX et al. (2025) | 2 | 1 | 1 | 2 | 1 | 0 | 2 | 0 | 2 | 2 | 2 | 2 | 17 | Medium quality |
| Shi P et al. (2025) | 2 | 1 | 1 | 2 | 1 | 0 | 2 | 0 | 2 | 2 | 2 | 2 | 17 | Medium quality |
| Liu Y et al. (2025) | 2 | 1 | 1 | 2 | 1 | 0 | 2 | 0 | 2 | 2 | 2 | 2 | 17 | Medium quality |
| Ke H et al. (2024) | 2 | 1 | 1 | 2 | 1 | 0 | 2 | 0 | 2 | 2 | 2 | 2 | 17 | Medium quality |
| Liu RN et al. (2024) | 2 | 1 | 1 | 2 | 1 | 0 | 2 | 0 | 2 | 2 | 2 | 2 | 17 | Medium quality |
| Yang FF et al. (2021) | 2 | 1 | 1 | 2 | 1 | 0 | 2 | 0 | 2 | 2 | 2 | 2 | 17 | Medium quality |
| Miao LH et al. (2019) | 2 | 2 | 1 | 2 | 1 | 0 | 2 | 0 | 2 | 2 | 2 | 2 | 18 | Medium quality |

**Appendix 5 The shapes of the funnel plots for the secondary outcomes of TS, LSE, LI, SLA, CTA, APS, and CTS**

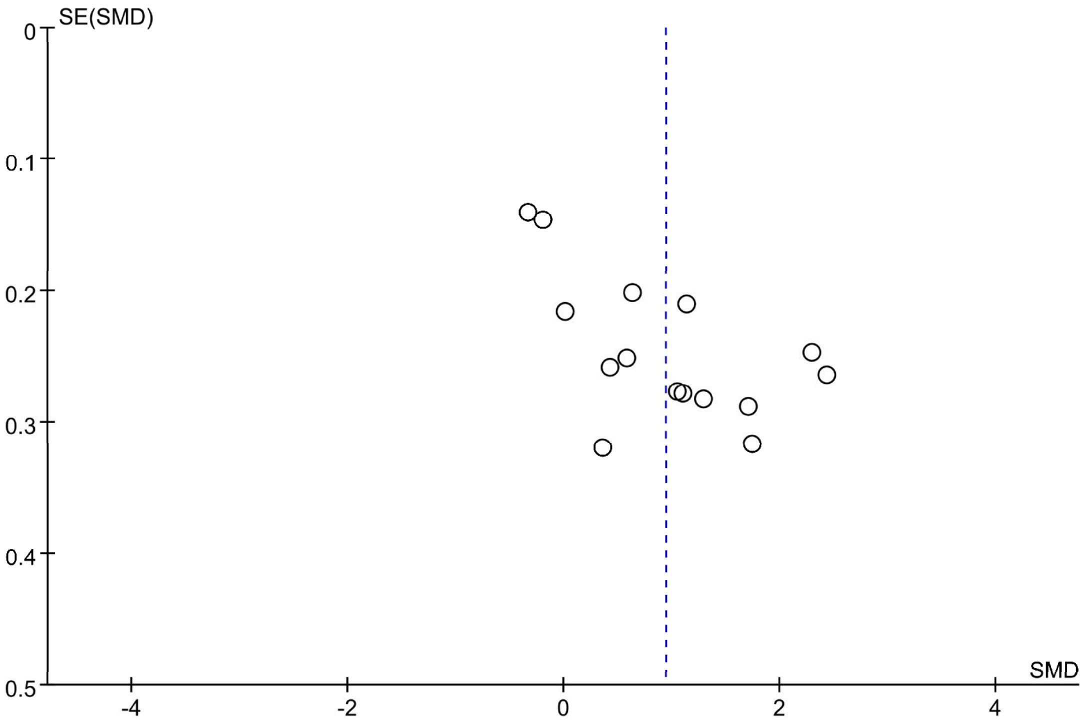


Figure 5.1 The shapes of the funnel plots for teaching satisfaction (TS) of continuous variables

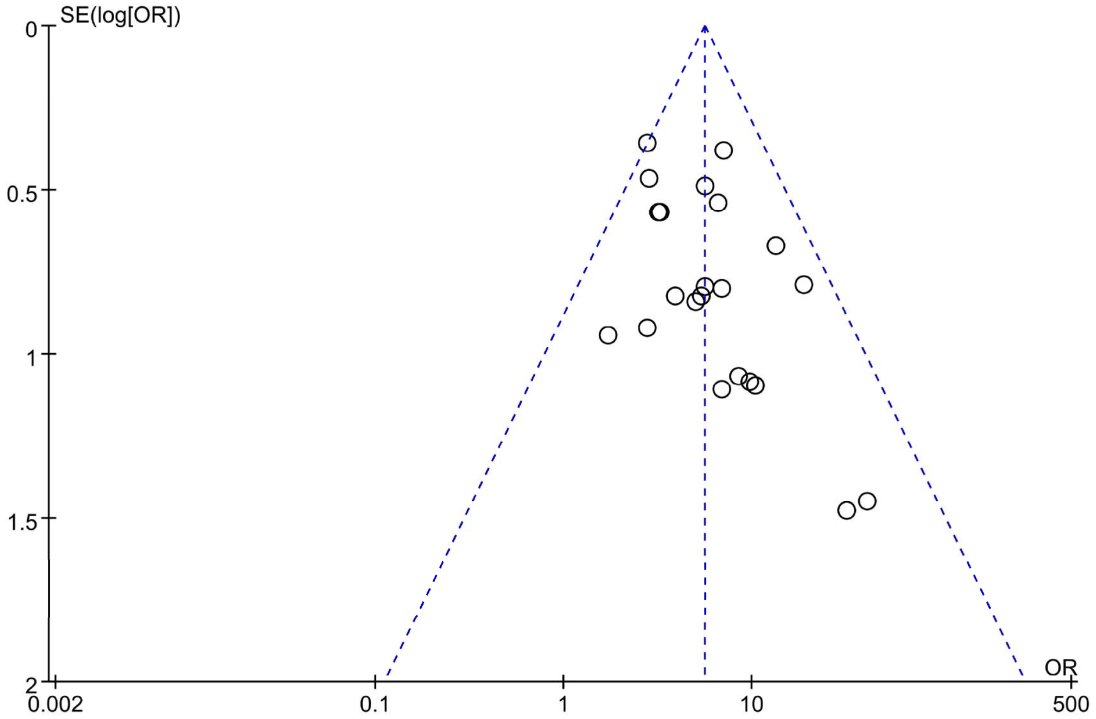


Figure 5.2 The shapes of the funnel plots for teaching satisfaction (TS) of dichotomous variables

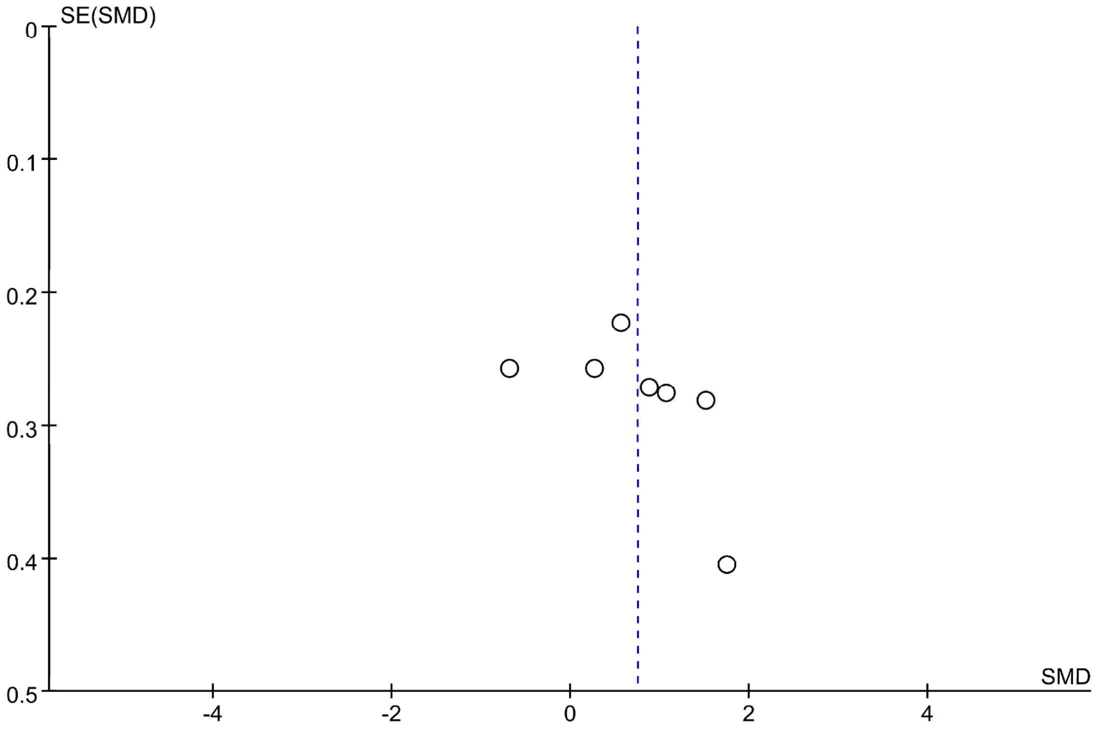


Figure 5.3 The shapes of the funnel plots for learning self-efficacy (LSE) of continuous variables

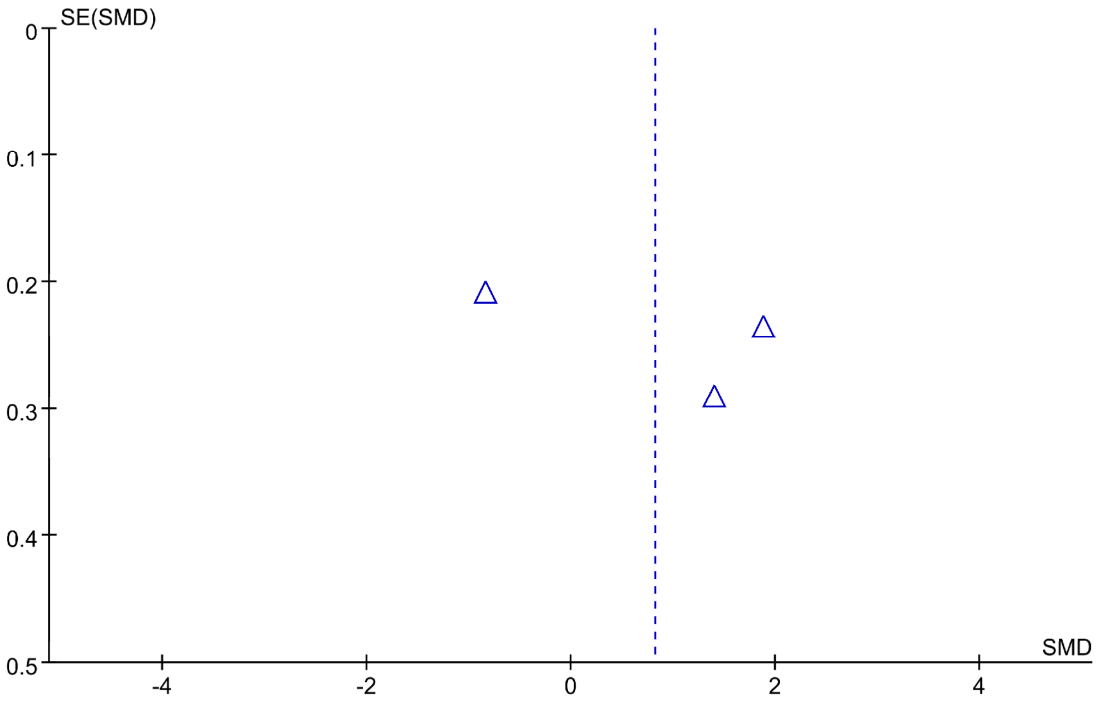


Figure 5.4 The shapes of the funnel plots for critical thinking skills (CTS) of continuous variables

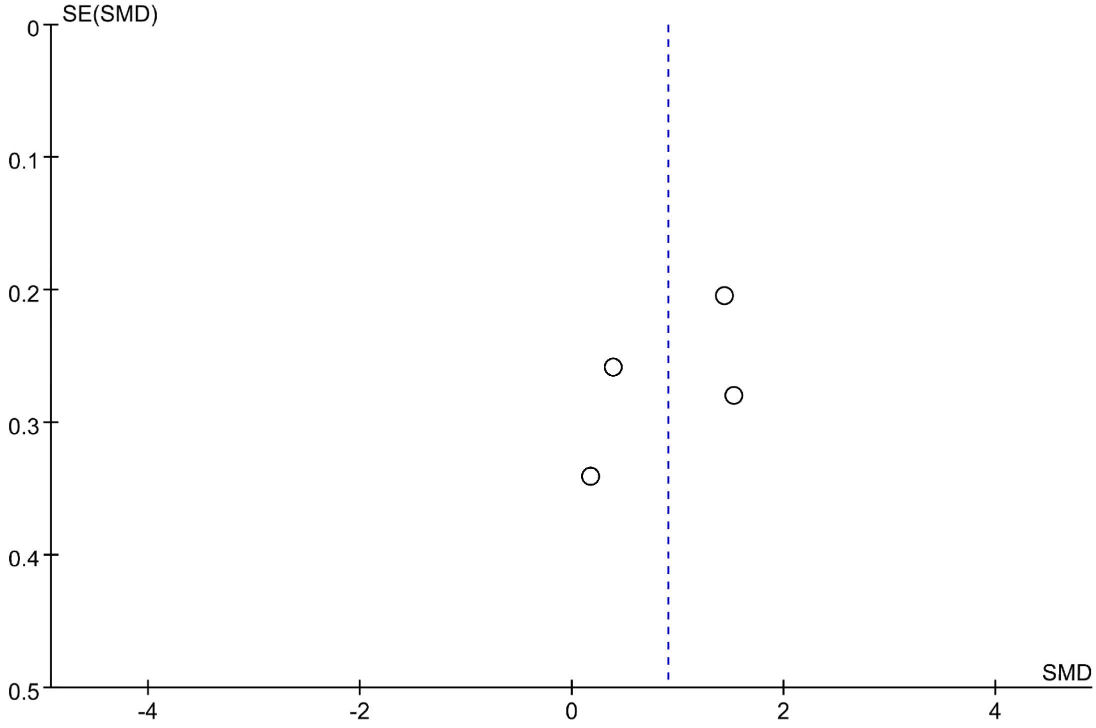


Figure 5.5 The shapes of the funnel plots for learning initiative (LI) of continuous variables

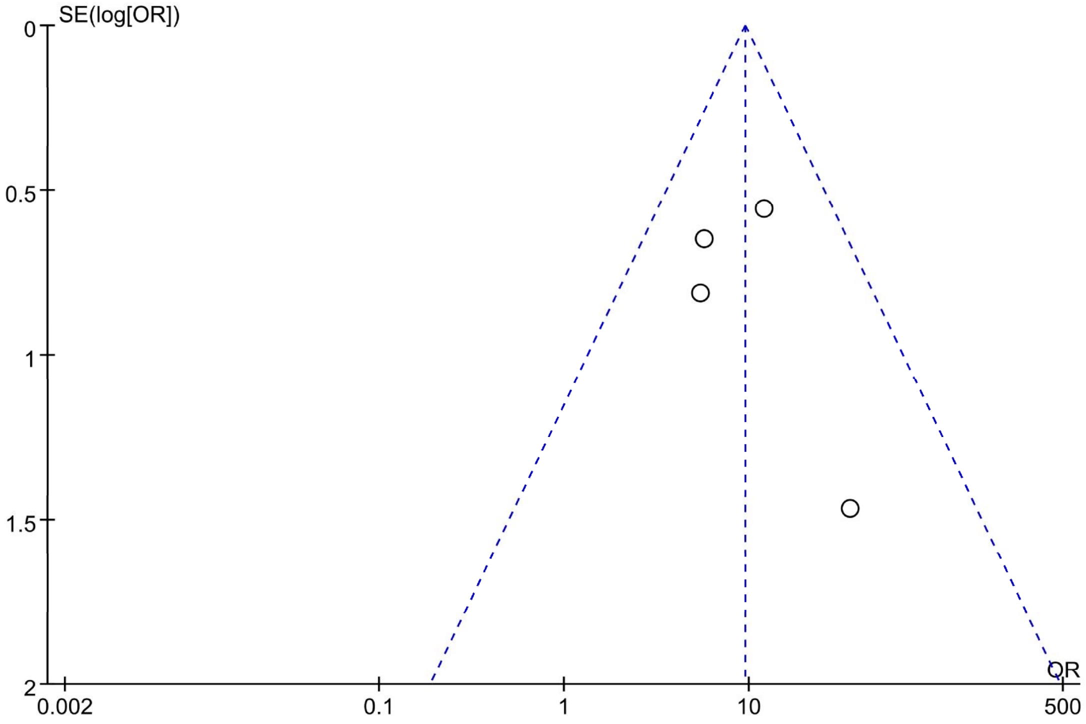


Figure 5.6 The shapes of the funnel plots for learning initiative (LI) of dichotomous variables

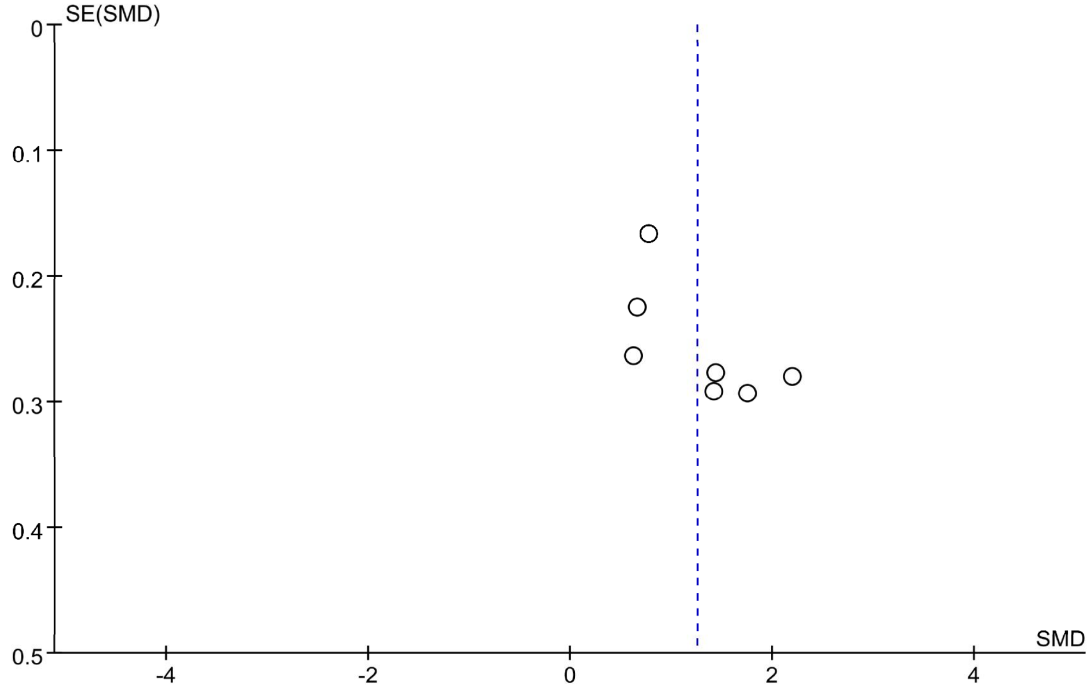


Figure 5.7 The shapes of the funnel plots for self-directed learning ability (SLA) of continuous variables

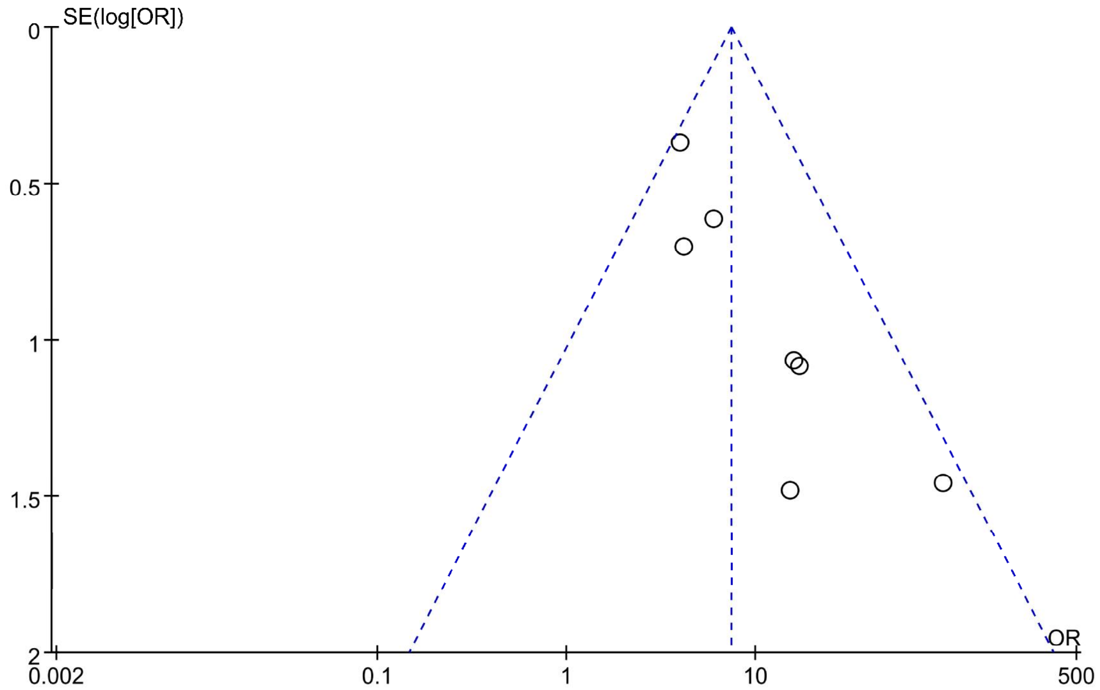


Figure 5.8 The shapes of the funnel plots for self-directed learning ability (SLA) of dichotomous variables

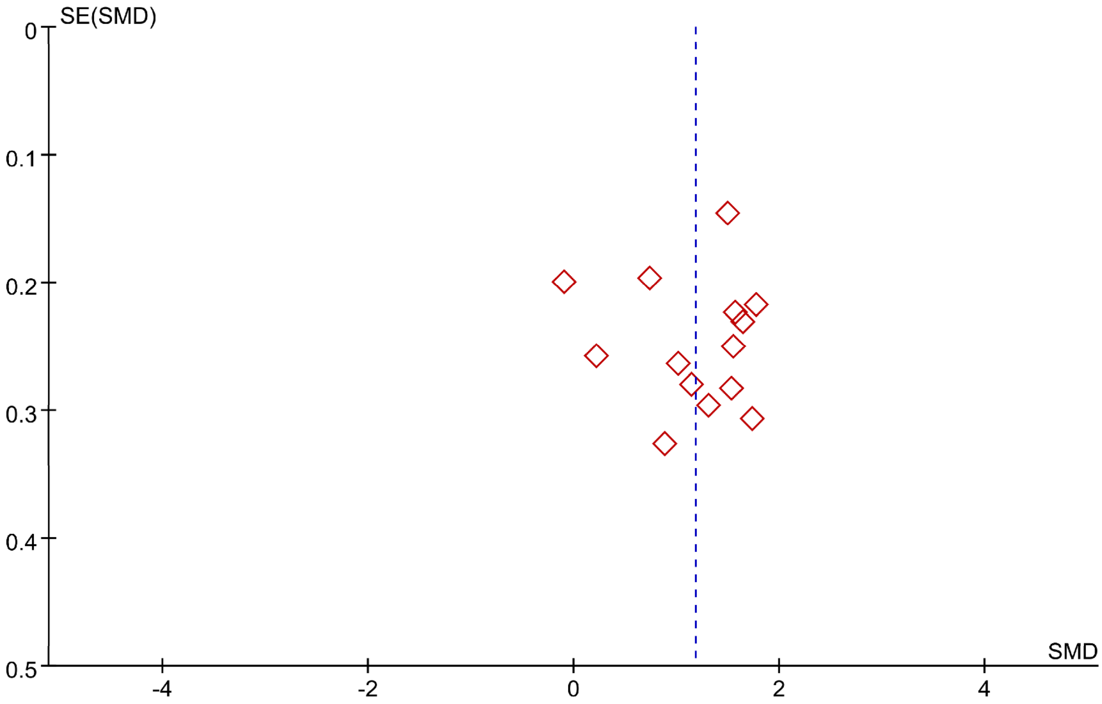


Figure 5.9 The shapes of the funnel plots for clinical thinking ability (CTA) of continuous variables

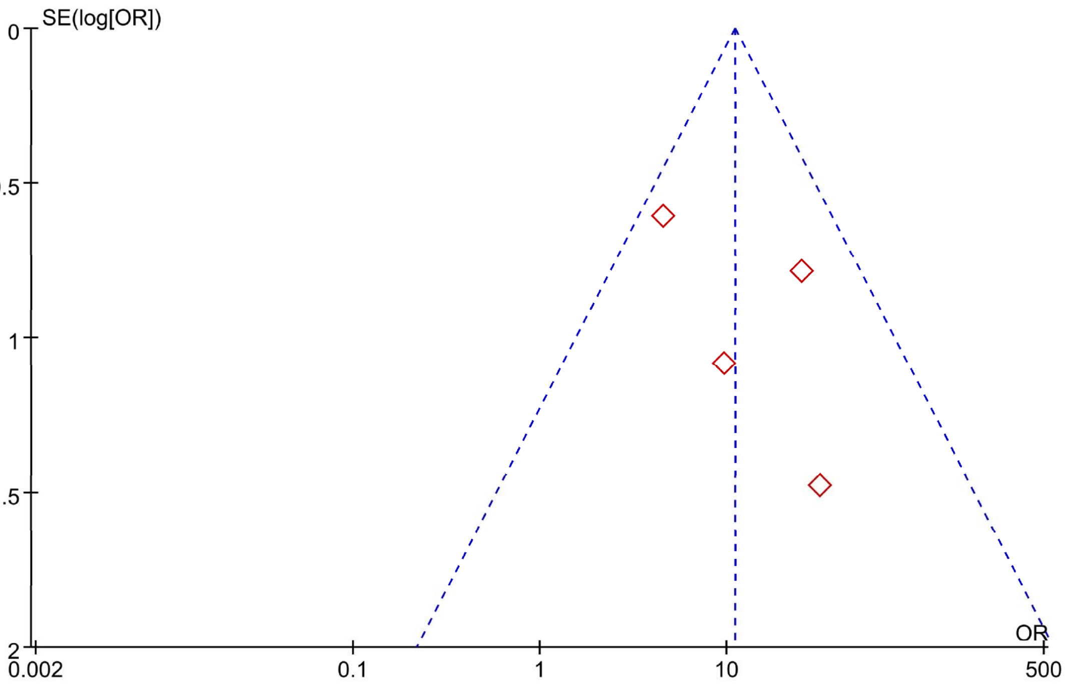


Figure 5.10 The shapes of the funnel plots for clinical thinking ability (CTA) of dichotomous variables

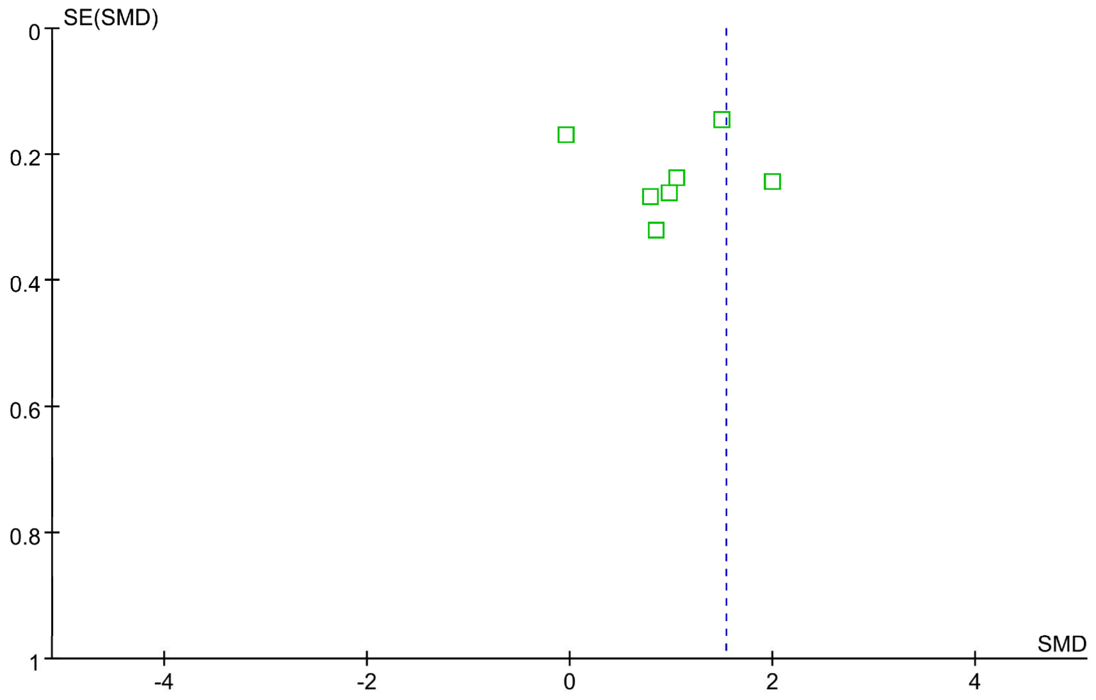


Figure 5.11 The shapes of the funnel plots for analytical and problem-solving skills (APS) of continuous variables

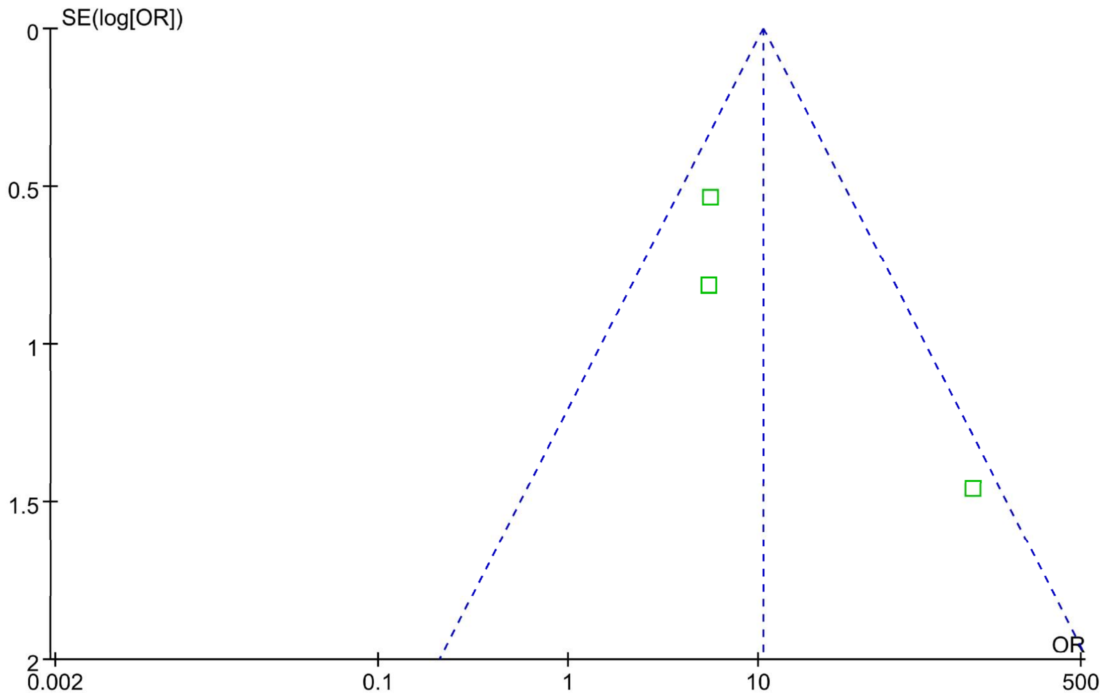


Figure 5.12 The shapes of the funnel plots for analytical and problem-solving skills (APS) of dichotomous variables

**Appendix 6 The sensitivity analysis using the one-study-removed method for primary outcomes**

**Table 6.1 The sensitivity analysis for the outcome of knowledge examination scores**

| Study omitted | Estimate | [95% Conf. | Interval] |
|---|---|---|---|
| Akutay S et al. (2024) | .97448712 | .73931001 | 1.2096642 |
| Al Kahf S et al. (2023) | .97688039 | .74060424 | 1.2131565 |
| Aneesh KV et al. (2025) | .9818449 | .74962476 | 1.214065 |
| Bai Y et al. (2024) | .96850099 | .73515979 | 1.2018422 |
| Bhatia AP et al (2024) | 1.0080324 | .79918013 | 1.2168847 |
| Cai W et al. (2023) | .93098486 | .7031824 | 1.1587873 |
| Cheng YX et al. (2025) | .96292022 | .73009713 | 1.1957433 |
| Chen Y (2025) | .96617683 | .73386208 | 1.1984916 |
| Coelho MS et al. (2025) | .98207919 | .75106105 | 1.2130973 |
| Cui XP et al. (2024) | .9433534 | .7134965 | 1.1732103 |
| Du YJ et al. (2025) | .93628235 | .7076402 | 1.1649245 |
| Ergezen Sahin G et al. (2025) | .95966167 | .72774233 | 1.191581 |
| Fan HX et al. (2025) | .97247507 | .73852974 | 1.2064204 |
| Feng M et al. (2024) | .96492029 | .73155462 | 1.1982859 |
| Fen LH et al. (2025) | .96013566 | .72772376 | 1.1925476 |
| Fernández-Alemán et al (2016) | .97283515 | .73787181 | 1.2077985 |
| Fu HR et al. (2025) | .9520452 | .72051513 | 1.1835753 |
| Gan W et al. (2024) | .97246273 | .73860471 | 1.2063207 |
| Han JW et al. (2023) | .98478358 | .75388429 | 1.2156829 |
| Han JW et al. (2025) | .97051337 | .73769855 | 1.2033282 |
| H?hne E et al. (2025) | .98207947 | .75067415 | 1.2134848 |
| Huang S et al. (2025) | .9816707 | .74940676 | 1.2139346 |
| Kalam KA et al.(2025) | .94150403 | .71143831 | 1.1715697 |

| | | | |
|---|---|---|---|
| Ke H et al. (2024) | .96863247 | .73529649 | 1.2019684 |
| Kejingyun S et al. (2025) | .9832908 | .75197463 | 1.214607 |
| Liang C et al. (2025) | .96482871 | .73082224 | 1.1988352 |
| Liang X et al. (2025) | .95102199 | .71956386 | 1.1824801 |
| Liaw SY et al. (2023) | .96688939 | .7339444 | 1.1998344 |
| Li L et al. (2024) | .96898953 | .73613953 | 1.2018395 |
| Li L et al. (2025) | .93894325 | .71006161 | 1.1678249 |
| Liu J et al. (2022) | .90997142 | .68937608 | 1.1305667 |
| Liu MD et al. (2025) | .95689482 | .72473208 | 1.1890576 |
| Liu RN et al. (2024) | .95721327 | .72458226 | 1.1898443 |
| Liu Y et al. (2025) | .94292634 | .71323661 | 1.1726161 |
| Mayor-Silva LI et al. (2025) | .98881553 | .76061297 | 1.2170181 |
| Molu Birsel (2025) | .94661706 | .71599366 | 1.1772405 |
| Qin W et al. (2025) | .95584478 | .72380584 | 1.1878837 |
| Roganovi? J (2024) | .97433232 | .74219122 | 1.2064734 |
| Shi P et al. (2025) | .96951986 | .73264695 | 1.2063928 |
| Tseng LP et al. (2025) | .96607425 | .73012605 | 1.2020224 |
| Wang D et al. (2025) | .97098615 | .73603173 | 1.2059406 |
| Wang DX et al. (2024) | .97313326 | .7399079 | 1.2063586 |
| Wang HY et al. (2025) | .94815626 | .71727622 | 1.1790363 |
| Wang X et al. (2022) | .93350906 | .70666971 | 1.1603484 |
| Wu C et al. (2025) | .94786915 | .71698886 | 1.1787494 |
| Yang FF et al. (2021) | .91588936 | .69092238 | 1.1408563 |
| Yu Z et al. (2025) | .95698512 | .72420633 | 1.1897639 |
| Zeng J et al. (2025) | .97679695 | .74304041 | 1.2105535 |
| Zhang ML et al. (2025) | .9720089 | .73878659 | 1.2052312 |
| Zhao C et al. (2023) | .97003786 | .73704373 | 1.203032 |
| Zhao Y et al. (2024) | .95547193 | .72344195 | 1.1875019 |
| Zheng K et al. (2024) | .94881466 | .71778068 | 1.1798486 |
| Zheng XS et al. (2025) | .95074318 | .71931493 | 1.1821714 |

| | | | |
|---|---|---|---|
| Zhong WY et al. (2024) | .96198169 | .72893616 | 1.1950272 |
| Zhu B et al. (2025) | .93637329 | .70929909 | 1.1634475 |
| Zhu DR et al. (2025) | .96113411 | .7284019 | 1.1938663 |
| Zhu YL et al. (2025) | .96380976 | .72881208 | 1.1988074 |
| Combined | .96142089 | .7320457 | 1.1907961 |

**Table 6.2 The sensitivity analysis for the outcome of practical examination scores**

| Study omitted | Estimate | [95% Conf. | Interval] |
|---|---|---|---|
| Bai Y et al. (2024) | 1.1442624 | 1.0750672 | 1.2134575 |
| Cai W et al. (2023) | 1.1503218 | 1.0811767 | 1.219467 |
| Chang CY et al. (2025) | 1.1776805 | 1.1080886 | 1.2472724 |
| Chun J et al. (2025) | 1.2006614 | 1.130812 | 1.2705108 |
| Cui XP et al. (2024) | 1.1445668 | 1.0753316 | 1.2138019 |
| Dupont V et al. (2025) | 1.170161 | 1.1007665 | 1.2395554 |
| Du YJ et al. (2025) | 1.1507087 | 1.0814402 | 1.2199771 |
| Ergezen Sahin G et al. (2025) | 1.1751856 | 1.1059064 | 1.2444647 |
| Feng C et al. (2025) | 1.153202 | 1.0821355 | 1.2242684 |
| Feng M et al. (2024) | 1.1608262 | 1.0911934 | 1.230459 |
| Fu HR et al. (2025) | 1.1601117 | 1.0907267 | 1.2294967 |
| Gao L et al. (2020) | 1.1583788 | 1.0885346 | 1.2282231 |
| Gao Y et al. (2025) | 1.1632453 | 1.093822 | 1.2326686 |
| Gokkurt Yilmaz BN et al. (2025) | 1.1427466 | 1.0731454 | 1.2123477 |
| Han JW et al. (2025) | 1.1667308 | 1.0972725 | 1.236189 |
| Hassoulas A et al. (2025) | 1.1694014 | 1.1002905 | 1.2385123 |
| H?hne E et al. (2025) | 1.1863556 | 1.1169018 | 1.2558094 |
| Huang S et al. (2025) | 1.1962419 | 1.1253608 | 1.2671229 |

| | | | |
|---|---|---|---|
| Hui Z et al. (2025) | 1.1692333 | 1.0999202 | 1.2385464 |
| Kang SL et al. (2023) | 1.1421633 | 1.0724621 | 1.2118645 |
| Ke H et al. (2024) | 1.1812143 | 1.1114715 | 1.250957 |
| Kestel S et al. (2025) | 1.1506861 | 1.081265 | 1.2201073 |
| Liaw SY et al. (2023) | 1.1857932 | 1.1161917 | 1.2553947 |
| Liaw SY et al. (2025) | 1.2171703 | 1.1466921 | 1.2876486 |
| Li L et al. (2024) | 1.1732212 | 1.1037119 | 1.2427305 |
| Liu MD et al. (2025) | 1.1675262 | 1.0980602 | 1.2369922 |
| Liu RN et al. (2024) | 1.1467103 | 1.0772391 | 1.2161815 |
| Liu X et al. (2025) | 1.1668997 | 1.0974143 | 1.236385 |
| Miao LH et al. (2019) | 1.1719224 | 1.1011354 | 1.2427095 |
| Qin W et al. (2025) | 1.1541652 | 1.0851873 | 1.2231431 |
| Simsek-Cetinkaya S et al. (2023) | 1.2308366 | 1.160935 | 1.3007382 |
| Uysal Yal??n S et al. (2025) | 1.198385 | 1.129076 | 1.267694 |
| Wang D et al. (2025) | 1.1810727 | 1.1106092 | 1.2515361 |
| Wang DX et al. (2024) | 1.1731861 | 1.1034509 | 1.2429214 |
| Wang HY et al. (2025) | 1.154323 | 1.0848155 | 1.2238304 |
| Wang X et al. (2022) | 1.1381012 | 1.0688134 | 1.207389 |
| Wang Z et al. (2025) | 1.1529267 | 1.0837249 | 1.2221285 |
| Wu C et al. (2025) | 1.1716351 | 1.1021273 | 1.241143 |
| Xu YM et al. (2021) | 1.1656228 | 1.0961746 | 1.235071 |
| Yamamoto A et al. (2024) | 1.1877154 | 1.1176732 | 1.2577577 |
| Yan CC et al. (2021) | 1.1703128 | 1.1006364 | 1.2399892 |
| Yang FF et al. (2021) | 1.1515513 | 1.0825242 | 1.2205785 |
| Yilmaz R et al. (2024) | 1.1579665 | 1.0886026 | 1.2273304 |
| Yu Z et al. (2025) | 1.1479425 | 1.078284 | 1.2176011 |
| Zhang ML et al. (2025) | 1.1812078 | 1.1114632 | 1.2509524 |
| Zheng K et al. (2024) | 1.1803077 | 1.1107042 | 1.2499112 |
| Zheng XS et al. (2025) | 1.1541811 | 1.0844672 | 1.223895 |
| Zhong WY et al. (2024) | 1.1677833 | 1.0981279 | 1.2374387 |

| | | | |
|---|---|---|---|
| Zhu B et al. (2025) | 1.1519474 | 1.0820866 | 1.2218082 |
| Zhu DR et al. (2025) | 1.1605851 | 1.0911176 | 1.2300525 |
| Combined | 1.1674073 | 1.0984922 | 1.2363225 |
